# Dynamic states of an MHC class I molecule during peptide exchange

**DOI:** 10.1038/s41467-026-77731-6

**Published:** 2026-09-10

**Authors:** Alexandra Mitlehner, Aldo S. Pasos-Trejo, Moritz Becker, Huan Lan, Lennard Berg, Tarek Hilal, Bernhard Loll, Benno Kuropka, Cecilia Clementi, Christian Freund

**Affiliations:** 1 Laboratory of Protein Biochemistry, Institute of Chemistry and Biochemistry, Freie Universität Berlin, Berlin, Germany; 2 Department of Physics, Freie Universität Berlin, Arnimallee 12, Berlin, Germany; 3 Research Center of Electron Microscopy, Institute of Chemistry and Biochemistry, Freie Universität Berlin, Berlin, Germany; 4 Laboratory of Structural Biochemistry, Institute of Chemistry and Biochemistry, Freie Universität Berlin, Berlin, Germany; 5 Core Facility BioSupraMol, Freie Universität Berlin, Berlin, Germany

**Keywords:** Cryoelectron microscopy, MHC class I

## Abstract

Antigens of intracellular origin are processed then presented by proteins of the highly polymorphic major histocompatibility complex I (MHCI), thereby enabling T cell activation during an immune response. In particular, the peptide-exchange catalyst tapasin (Tsn) plays a critical role in shaping the pool of peptide antigens that ultimately reach the cell surface. Here, using disulfide bond engineering in conjunction with cryogenic electron microscopy (cryoEM) and molecular dynamics simulations, we provide evidence for partial collapse of the antigen binding groove during the peptide exchange process. An intermediate is formed that is characterized by interactions between the peptide’s N-terminus and conserved tyrosine side chains in MHCI. Unfolding of the MHCI α1-helix in the Tsn-bound state is contrasted by a stable α2-helix that is kept in its native-like major conformation by the support of the large interface it entertains with the exchange catalyst. Helical disorder propensities and backbone flexibilities of the α1-helix are predicted to have increased during evolution, suggesting that the dynamic features introduced by polymorphic variation may have contributed to shaping the pool of antigens presented to T cells.

## Abstract (web summary)

Tapasin is a protein that catalyzes the exchange of low-affinity peptides in the MHC-I peptide-binding groove for high-affinity ones. Here, the authors show that peptide exchange proceeds through an MHC-I intermediate characterized by unfolding of the α1-helix and anchoring of the peptide N-terminus at the tapasin-stabilized α2 helix.

## Introduction

Major Histocompatibility Complex class I (MHCI) proteins present antigens for CD8^+^ cytotoxic T cells on nucleated cells. The presented pool of antigens originates from intracellular (self-)proteins for immunosurveillance, enabling the detection of infected or tumorigenic cells by the immune system and subsequent elimination of target cells^1–3^. The process of peptide loading and editing for MHCI is taking place at the luminal side of the endoplasmic reticulum membrane. Here, the peptide loading complex (PLC) is assembled to load high-affinity peptides onto MHCI proteins. The PLC consists of the transporter associated with antigen processing (TAP), the chaperone tapasin (Tsn) and the oxidoreductase ERp57, as well as calreticulin (Crt). Tsn is able to catalyze the peptide exchange of suboptimal peptides bound on MHCI in favor of high-affinity antigens, leading to an edited pool of peptides presented on the cell surface^4–10^. The second quality control of peptides takes place outside of the PLC and is provided by TAPBPR (TAP-binding protein related), which can also catalyze the exchange of peptides on MHCI proteins and binds in a similar way to MHCI^11–13^. Despite these mechanisms, there appear to be peptides presented on MHC I that display relatively low affinity and which nevertheless elicit strong T-cell responses^14^.

Two single-particle cryoEM structures of the entire PLC^4,15^, as well as crystal structures^16,17^, have provided insight into the interaction sites of MHCI with Tsn. These structures show the editing loop of Tsn positioned above a fully formed F-pocket region of MHCI, where the C-terminal part of the peptide binds. Tsn binding, therefore, is thought to be incompatible with a peptide binding to the F-pocket, as the L18 residue of Tsn’s editing loop partially occupies the space where αY84 of MHCI is positioned to form a hydrogen bond with the C-terminus of a bound peptide. While all previously published structures of empty MHCI bound to Tsn or TAPBPR show a fully formed peptide binding groove, a recent study from Sun et al. revealed that TAPBPR-bound and suboptimally loaded MHCI leads to a certain degree of flexibility in the α1-helix, indicating that the actual process of peptide exchange and editing might include dynamics that were not represented by the previous structures^18^. Thus, the available cryoEM and crystal structures appear to represent snapshots that describe certain features of the editing process, but it remains elusive to understand how the conformational interplay between MHCI, peptide and Tsn choreographs the exchange process.

To further investigate this dynamic process of peptide editing, we used disulfide-engineered MHCI and Tsn variants to determine the malleability of the interaction between Tsn’s editing loop and the α1-helix of MHCI in the presence of a suboptimal peptide. Cysteines at positions 12, 16 and 18 in Tsn’s editing loop are all able to form disulfide bridges to the positions α84, α83 and α73 in the α1-helix of A*02:01, indicating conformational flexibility. We show that these mutant complexes are still fully functional in vitro, and we analyze the structure of the human HLA-A*02:01 (HLA-A2) αY84C variant disulfide-bridged to Tsn K16C. CryoEM analysis reveals several states for which the α1-helix is only transiently resolved, fluctuating between an unfolded and a folded state. The peptide is bound independently of α1-folding by contacting conserved residues in the β-sheet floor and the α2 domain. We suggest that peptide dissociation and association progress through a state where the Tsn-stabilized α2-helix provides the landing and leaving platform for the peptide, while the α1-helix potentially acts as a flexible entry or exit gate within the larger peptide loading complex.

## Results

### Disulfide bond formation between tapasin editing loop and the MHCI α1-helix

**Fig. 1:**
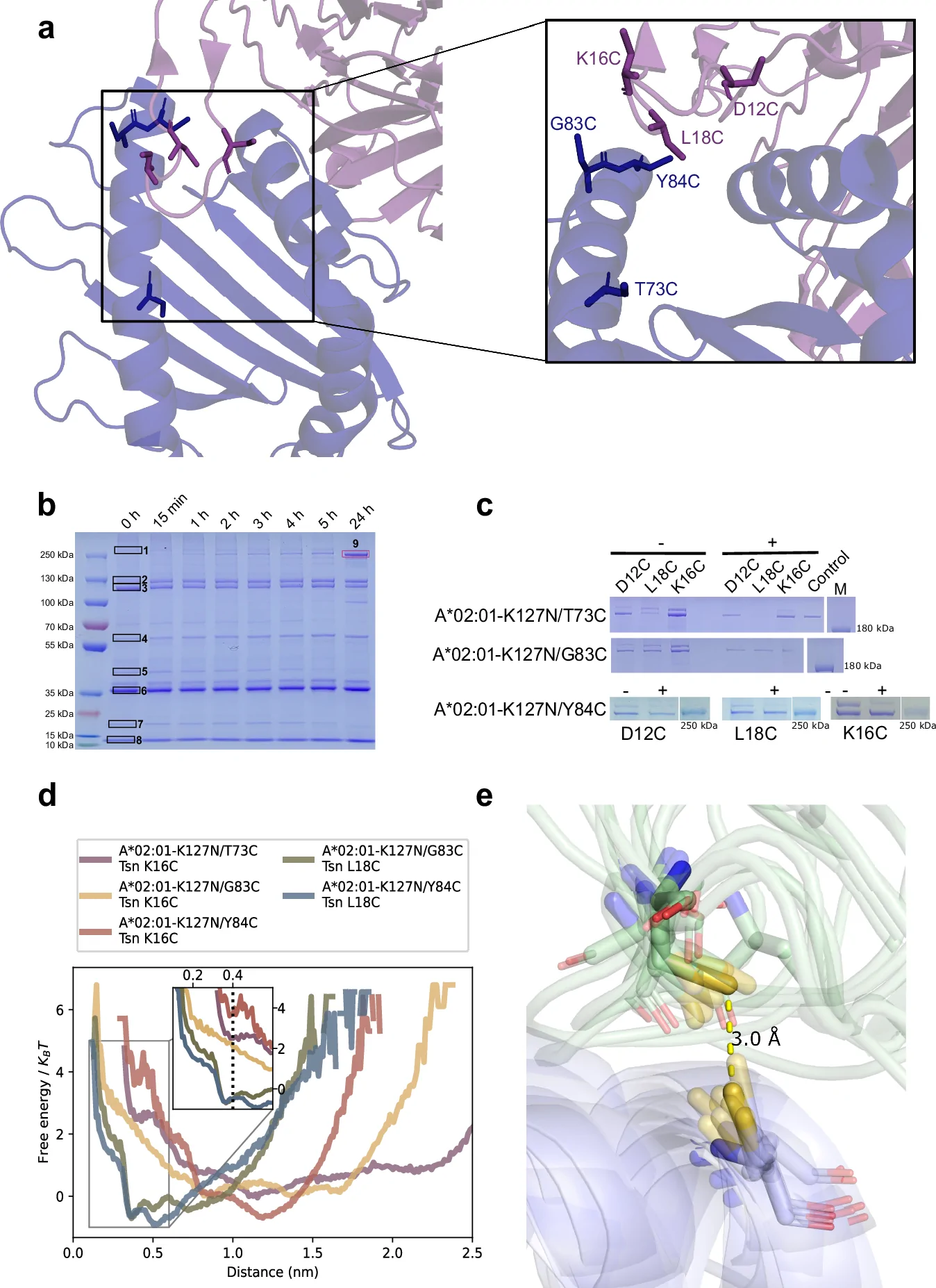
Disulfide-bond formation reports on the dynamics of the MHCI binding groove. **a** Top-view of the upper interaction interface between MHCI (blue) and Tsn (purple). Residues that were mutated to cysteine are shown as sticks. (modified from PDB ID: 7QPD^4^). **b** Complex formation kinetics for A2-αY84C with Tsn-K16C. Annotated bands are: 1-Tsn/ERp57 multimer, 2-Tsn/ERp57-β_2_m, 3-Tsn/ERp57, 4- ERp57, 5-Tsn, 6-A2-heavy chain, 7-β_2_m dimer, 8-β_2_m, 9- disulfide-bridged A2-αY84C/Tsn-K16C complex. **c** Complex formation gels of A2-αY84C, αT73C, and αG83C with Tsn-D12C, K16C and L18C. Addition of redox buffer (GSH/GSSG) is indicated with a +. Control contains A2-αY84C/Tsn-K16C complex with redox buffer. The lower visible band indicates successful complex formation, while the upper band contains a Tsn/ERp57 multimer. Shown is the part of the gel containing bands 1 and 9 from (**b**). Full gels are shown in Supplementary Fig. 2. The experiment was done twice for A*02:01-K127N/Y84C and once for A*02:01-K127N/T73C and A*02:01-K127N/G83C. **d** Free energy profile of S-S distances from MD simulations of the indicated mutations. The black dashed line at 4 Å highlights the threshold for contacts consistent with the formation of a disulfide bridge. **e** Representative ensemble of ten A2-αY84C, Tsn-D12C structures with close S–S contacts as sampled by molecular dynamics (MD) simulations. The starting point for the simulations is complexes without formed disulfide bonds. Source data are provided as a Source Data file.

The current structures of peptide-MHCI (pMHCI) in complex with Tsn suggest the editing loop of the exchange catalyst to flexibly hover on top of the F-pocket^4,16^. It is thought that this loop competes with the C-terminus of the peptide, with the side chain of αY84 having disengaged from its hydrogen bond to the terminal carboxylate of the peptide and instead forming an interaction with E72 of Tsn. Concomitantly, the binding groove widens by a few Å when bound to the exchange catalyst, while L18 of Tsn partially occupies the space formerly assumed by αY84. Whether additional conformational changes are operative along the exchange pathway is not known. Thus, we decided to probe conformational flexibility of the two molecules by introducing disulfide bonds at distinct positions. For Tsn, we focused on the editing loop with residues D12, K16, and L18 mutated to cysteine. For the heavy chain of the MHCI HLA-A2 molecule we mutated conserved residues along the α1-helix, namely αY84C, αG83C and αT73C (Fig. 1a). In addition, we implemented a T5C mutation in β_2_m and G304C in Tsn to allow a covalent bond to be formed independent of the editing loop, thus not limiting complex formation by the low affinity of the interaction as well as a K127N mutation in HLA-A2, which is present in the more Tsn-susceptible HLA-A*03:01^7^. ERp57 contained a C60A mutation that was shown to trap the Tsn-ERp57 dimer as a complex, by forming a mixed disulfide^19,20^. Given the competitive binding of Tsn and peptide, we initiated complex formation by photo-cleaving the peptide (pKV9) and allowing the reaction to proceed under defined conditions. Subsequently, the reaction was subjected to SDS gel electrophoresis, and the correct complex band was exemplarily confirmed for the A*02:01-αK127N/αY84C complex by mass spectrometry to contain the MHCI heavy chain, β2m-T5C, Tsn-G304C/K16C and ERp57-C60A (Supplementary Fig. 1). Figure 1b, c and Supplementary Fig. 2 show the results, indicating that αT73C, αG83C, and αY84C are able to form disulfide bonds to all cysteine variants of the editing loop. For the αY84C/K16C complex, the kinetics of disulfide bond formation were monitored, indicating that a time of >5 h is required for the reaction to complete (Fig. 1b).

The finding that all combinations of disulfide bond pairings are able to form is unexpected, given that the Cβ (Cα in case of G83) atoms of the two bond-forming residues in the structure of the PLC (PDB ID: 7QPD^4^) are between 4.3 and 17.8 Å away from each other.

To assess whether the dynamics of the interface would indicate conformational changes that are compatible with disulfide bond formation, we performed molecular dynamics (MD) simulations with the corresponding mutants. We therefore investigated whether the cysteines would get close in space without introducing the disulfide bridge as a constraint, potentially allowing the disulfide bridge to be formed. The results are shown in Fig. 1d, e, indicating that the mutants can form disulfide bonds with distinct propensities. For example, HLA-A2-αY84C/Tsn-L18C showed a prominent fraction of conformations that would bring the two cysteine sulfur atoms into a proximity of less than 4 Å. For other mutants (e.g. HLA-A2-αY84C/Tsn-K16C or HLA-A2-αT73C/Tsn-K16C) such a close distance was a very rare event (Fig. 1d, e and Supplementary Fig. 3), yet it could occur at the time scale of microseconds (see Supplementary Table S1).

The slow kinetics of disulfide bond formation measured experimentally are in agreement with the few events of approximation observed theoretically. Both experimental and theoretical results thus show that disulfide bond compatible conformations can occur on rare occasions, highlighting the plasticity of the α1-helix in case peptide is (partially) unbound.

### Peptide exchange properties are compatible with cysteine mutations and disulfide bond formation

**Fig. 2:**
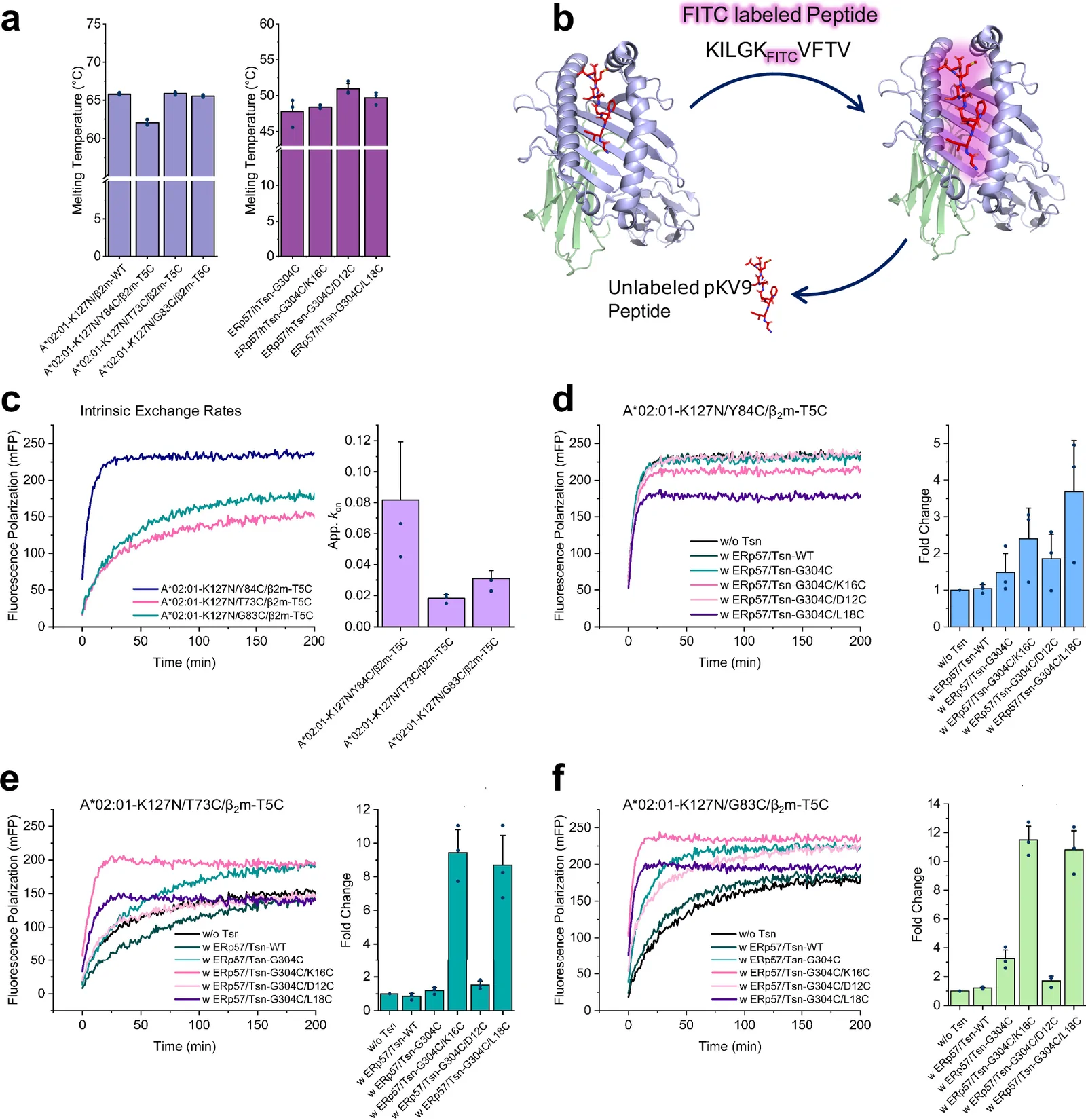
Biochemical studies of pMHCI and ERp57-C60A/Tsn cysteine variants. **a** Melting temperatures of pMHCI and Tsn cysteine mutants. Melting curves are shown in Supplementary Fig. 4. **b** Schematic of the FP-Assays to measure peptide exchange of MHCI cysteine mutants preloaded with photocleavable peptide (pKV9; KILGFVFJ*V). In our experiments we echange pKV9 against FITC-labeled KV9 (KILGK_FITC_VFTV) with or without ERp57-C60A/Tsn wt and variants at 25 °C. For the scheme the structure of HLA-A*03:01 in complex with the peptide AIFQSSMTK (PDB ID: 3RL1^70^) was chosen as a surrogate since the structure of HLA-A*02:01 with the KV9 peptide is not known." (.** c**–**f** Exemplary and cumulative results of the exchange kinetics of individual MHCI cysteine mutant proteins in the presence and absence of the different Tsn variants. Fold changes were calculated for each measurement relative to the apparent *k*_on_ value from the measurement without Tsn. Bar charts show the mean with standard deviation of three individual experiments with three in-plate replicates each. Source data are provided as a Source Data file.

To exclude the possibility that disulfide bond formation leads to global unfolding of the two proteins, we first performed biochemical experiments to probe the intactness of the molecules. Figure 2a shows that all investigated MHCI cysteine variants form thermodynamically stable complexes well above 37 °C when bound to the high-affinity peptide KILGFVFTV. The denaturation melting point (Tm) of most variants is comparable to that of the respective MHCI variant without a cysteine mutation in the α1-helix. The measured Tm of Tsn cysteine-variants is comparable to wt, which our group previously published to be at 49.3 °C^7^. MHCI αY84C has a Tm value of 4 °C lower than the other two variants (62 °C and 66 °C, respectively), indicating that the hydrogen bond of αY84 with the C-terminus of the peptide substantially stabilizes the MHCI-peptide complex. To monitor the ability of the respective variant complexes to exchange peptides, replacement of a placeholder peptide (KILGFVFJ*V) against the fluorescently-labeled high-affinity peptide (KILGK_FITC_VFTV) for HLA-A2 was monitored by fluorescence polarization (Fig. 2b). Comparing the three MHCI proteins under investigation, the αY84 variant showed a higher intrinsic exchange rate, again indicating that spontaneous peptide release is enhanced in this mutant (Fig. 2c). Interestingly, all MHCI variants display enhanced exchange rates in the presence of cysteine-mutated Tsn (Fig. 2d–f), especially for the αT73C and αG83C MHCI variants with Tsn-K16C and L18C. The rate enhancement is more pronounced when K16 and L18 in Tsn are replaced by cysteine. These two residues were previously shown to be critical for rate enhancement of allotypes with an acidic F-pocket (HLA-A3). Interestingly, the observed acceleration rather than attenuation of peptide exchange for HLA-A2 when mutating these two sites indicates that the Tsn editing loop is not optimized for this allele.

### Structural investigations on MHCI-Tsn complex reveal MHCI binding groove dynamics

Given the functional integrity of the disulfide-bonded MHCI-Tsn complexes, we then assessed the conformational rearrangements that would enable formation of the respective complexes. According to the biochemical data, the complex forming a disulfide bond between Tsn-K16C and HLA-A2-αY84C can be formed almost quantitatively, and the proteins could be expressed and purified in amounts sufficient for structural analysis by cryoEM. Complex formation was achieved by photocleaving the bound nonamer peptide (pKV9), adding the Tsn/ERp57 dimer and running a size exclusion chromatography column prior to concentration and application to the TEM grid (Supplementary Figs. 5 and 6a, b).

**Fig. 3:**
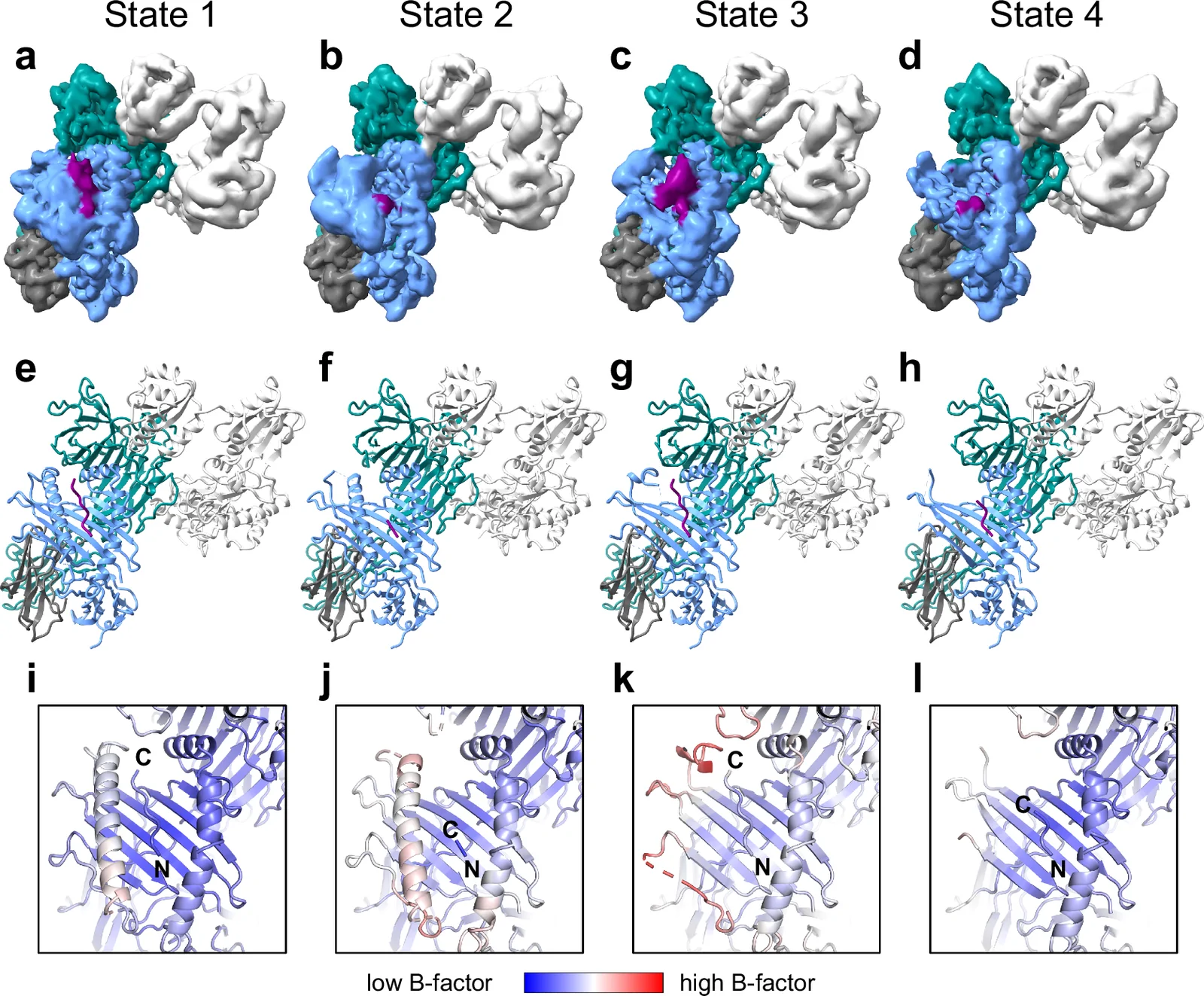
Structures of the disulfide-bridged variant pMHCI-Tsn/ERp57 complex. 3D classification of the cryoEM data revealed distinct structural states. The cryoEM reconstructions are shown in **a**–**d** with the corresponding models in **e**–**h** (state 1–4, respectively). ERp57 is depicted in white, MHCI heavy chain in light blue, Tsn in teal, β_2_m in dark gray and the peptide in purple. Specifically, in state 1 the helix is formed and the peptide is bound. In state 2 the helix is formed and peptide is largely dissociated. State 3 is missing a folded helix, but the peptide is bound. In state 4, the helix is missing, and the peptide is largely dissociated. The Tsn loop can be fully traced except for state 2. **i**–**l** Close up on the peptide binding groove of the states in **e**–**h** with cartoon representations colored according to the corresponding B-factors of each structure.

Initial cryoEM analysis of the complex resulted in poorly defined density within the peptide binding groove (Fig. 3a–d). Further classification by 3D variability analysis revealed heterogeneity in the peptide binding site (Supplementary Figs. 7 and 8), represented by a continuum of structural states between stably folded α1-helix and fully unfolded α1-helix (Supplementary Movie 1). We have selected the four most prominent classes regarding differences in density in the peptide binding groove and refined them individually to global resolutions between 3.5–3.6 Å (Table S2), highlighting differences in the α1-helix, the Tsn editing loop and the peptide (Fig. 3 and Supplementary Fig. 6c–n).

The most prominent differences between the classes represented in Fig. 3 are twofold: on the one hand, the peptide has dissociated to different degrees out of the binding pocket, and on the other hand, the α1-helix of the MHC heavy chain is unfolded to different degrees. The two events seem to be able to occur independently, as for example in state 2, the helix is largely formed (Fig. 3b), while only the first two amino acids of the peptide can be unambiguously traced. In contrast, while the α1-helix is fully unfolded, peptide density can be assigned to the 7mer peptide in state 3 (Fig. 3c). This observation does not exclude that both events also take place simultaneously, as, for example, state 3 shows the helix to be absent from residue α53 to α80 and the peptide to be partially dissociated from the groove. Interestingly, the editing loop of Tsn is well defined in three of the four states, indicating that its mobility is more restricted compared to the malleability of the α1-helix, which might be due to the disulfide bond holding it in place. In state 1, the α1-helix is fully visible, and the disulfide bond is formed (Fig. 3a), indicating that helix formation and the engineered cysteine bridge are compatible. Nevertheless, the most populated state based on its relative particle abundance in the full dataset is state 3 where the helix is not formed (Supplementary Fig. 8). Thus, we conclude that once peptide dissociation from its C-terminus is progressing, the region αE58-αG83 becomes a structural element of the MHCI molecule that exerts a high degree of conformational dynamics, with helix formation still occurring in a prominent fraction of molecules. However, it has to be noted that the B-factors for the states where the α1-helix can be modeled, are high, as indicated in the corresponding structures in Fig. 3i–l and Supplementary Fig. 7a–d. In contrast, the α2-helix is fully formed in all states with relatively low B-factors. In line with this B-factor distribution, the local resolution varies significantly for different parts of the complex, with the α1-helix and C-terminal part of the peptide displaying a resolution of around 5 Å, while it is below 3 Å for parts of the α2-helix (Supplementary Fig. 7e–h). Furthermore, when we estimate the Free Energy Surface (FES) for state 1 of the complex with an explicitly restricted disulfide bridge using atomistic molecular dynamics and a Markov-State model (MSM), we observed several different forms of unfolding/flexible movement of the α1-helix upon peptide dissociation (Supplementary Fig. 9). Also, the large interface between the α2-helix and Tsn is almost unaltered between states and the lower domain disulfide bond introduced to block dissociation of the complex expectedly does not lead to structural changes (Supplementary Fig. 10).

### Defining an intermediate of peptide binding

**Fig. 4:**
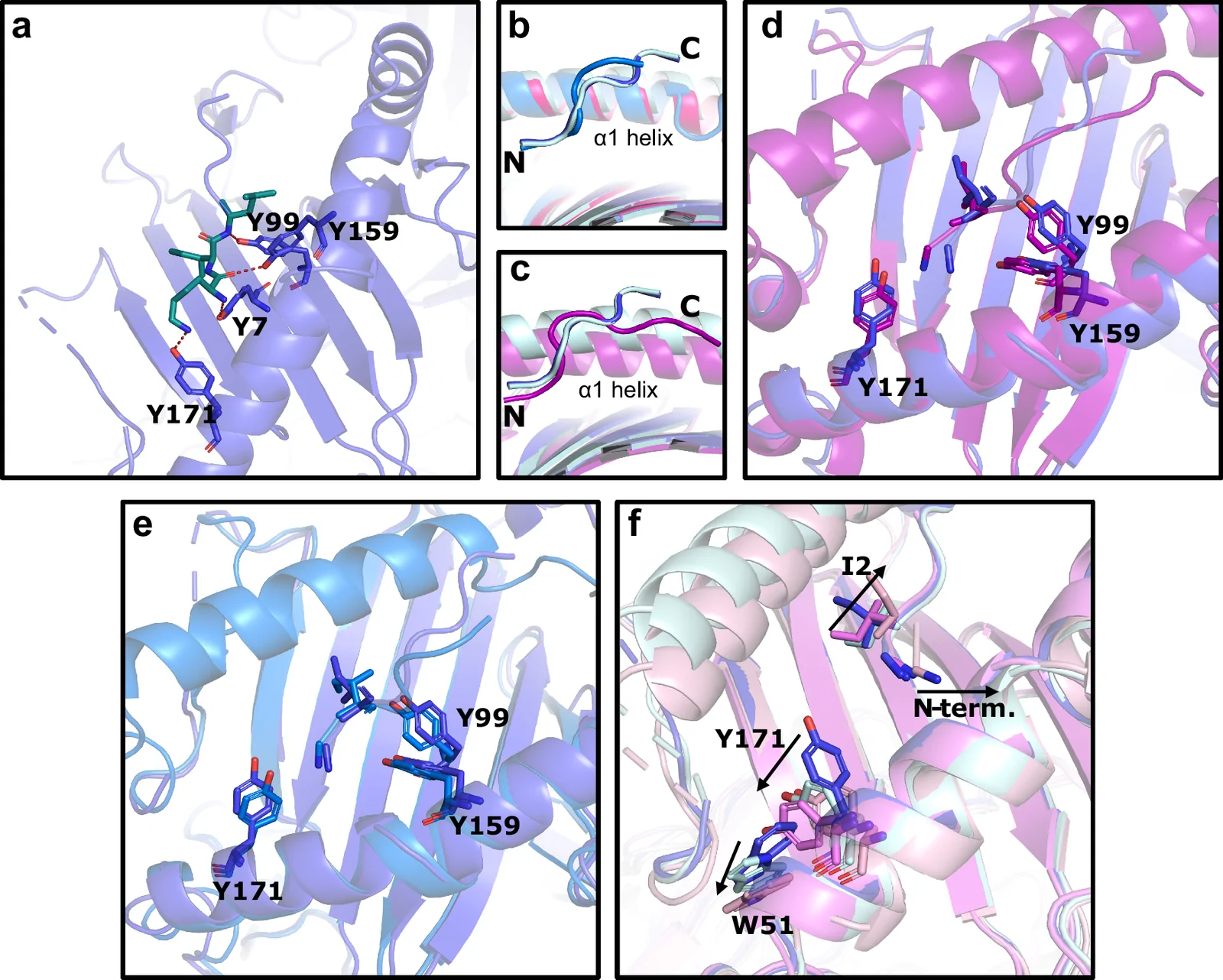
Close-up of the peptide-binding groove of state 1 and 3 in comparison to peptide-bound structures of MHCI, as well as in complex with TAPBPR or as part of the PLC. **a** Close-up of state 3 with focus on the first three residues of the 7-mer peptide (KILGFVF, colored in teal), αY7, αY99, αY159 and αY171 of A*02:01, as well as the peptide, is shown as sticks. **b** State 1 (light cyan) and 3 (blue) aligned with A*02:01-αG120C bound to the same 7-mer (PDB ID: 9C96^18^) in marine and a cryoEM structure of the PLC (PDB ID: 7QPD^4^) in pink. **c** Overlay of state 1 (light cyan) and 3 (blue) with a crystal structure of A*02:01 (PDB ID: 5HHP^29^) bound to the influenza peptide GILEFVFTL in purple. **d** State 3 (blue) aligned to a crystal structure of A*02:01 (PDB ID: 5HHP^29^) in purple, displaying the side chains of αY99, αY159 and αY171 and the N-terminal NH group and the side chain of peptide residue P2 (Ile). **e** Overlay of state 3 (blue) with A*02:01-αG120C (marine) bound to the same 7-mer (PDB ID: 9C96^18^) with the same highlighting of residues as in (**d**). **f** Conformational changes at the N-terminal binding site in the four states observed in this study (state 1 in pale cyan, state 2 in rosé, state 3 in blue and state 4 in violet).

Notably, all structural states we observe are at or near the thermodynamic equilibrium since the complexes between MHCI, peptide and Tsn-ERp57 were purified through size exclusion chromatography and concentrated subsequently. Thus, they represent potential states of either dissociating or associating peptide in a reversible, non-covalently bound state. Anchorage of the N-terminus of the peptide is seen in all of our structures, independent of whether the α1-helix is formed. Critical contacts are observed between conserved tyrosine residues of the β-sheet floor (αY99) or the α2-helix (αY159, αY171) and the peptide backbone of residues 1–3 (Fig. 4a), defining a tyrosine cradle prone to interact with the N-terminal part of the peptide in a conserved manner. Indeed, when comparing our state 3 structure with those of a stable peptide-HLA-A2 complex, we observe a very similar backbone and side chain configuration in the N-terminal peptide binding site (Fig. 4b, d). Moreover, this interaction site also superimposes well with the structure of the TAPBPR-bound HLA-A2 complex bound to the same 7mer peptide as reported here (Fig. 4c, e), which indicates conserved peptide recognition for the two chaperones. Notably, the α1-helix is not resolved in our state 3 structure, suggesting that the peptide-α2-helix interaction mode is preserved even when the α1-helix is not in a defined conformation.

Despite the overall similarities of the four states, we also observed distinct differences at the site of peptide binding. αY171 entertains a hydrogen bond to the N-terminal amino group of the peptide only in state 3, while it moves away in the other structures (Fig. 4f). Simultaneously, αW51 makes place for the αY171 side chain, and the N-terminal amino group is moving away from its hydrogen-bonded position in the groove. The side chain of the P2 residue also moves upwards, coinciding with the movement of the N-terminus. Thus, we have defined conserved and dynamic features of the antigen recognition process of MHCI molecules.

### Flexibility of the α1 domain as an evolutionary feature

**Fig. 5:**
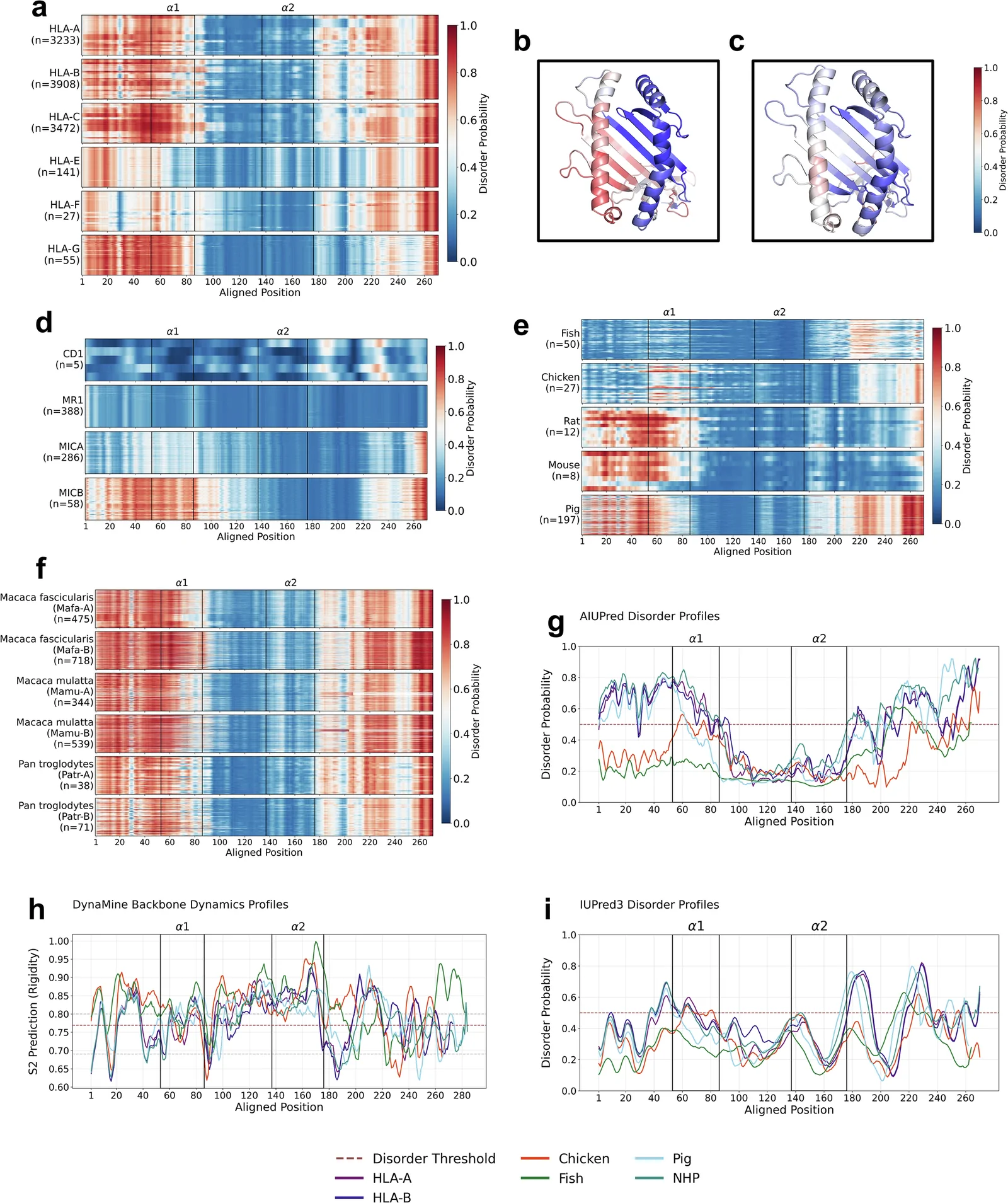
Predicted disorder and dynamics profiles for classical and non-classical HLA molecules and MHCI proteins derived from animals. Sequences were obtained from IPD-IMGT/HLA^56,57^, IPD-MHC^58^ or UniProt^59^ (CD1, MR1 and mouse sequences). For AIUPred and IUPred3, values over 0.5 indicate a disordered region. **a** Disorder probability values predicted with AIUPred for HLA-A,B,C,E,F and G. The sequence sections for α1- and α2-helix are marked with black boxes in all heatmaps. **b**, **c** Show the average disorder probability of HLA-A and B predicted with AIUPred and IUPred3, respectively, mapped onto an HLA-A*02:01 structure (PDB ID: 5HHP^29^). The peptide was removed to ensure better visibility of the binding groove. **d** AIUPred disorder predictions for non-classical HLA molecules (CD1, MICA, MICB and MR1). **e** AIUPred^22^ disorder predictions for animal-derived MHCI classes that are not part of the non-human primates (NHP) group. **f** AIUPred disorder predictions for selected NHP MHCI classes. **g** AIUPred disorder profiles as averages for HLA-A and B, fish, chicken, pig, and NHP (averaged over all analyzed MHC sequences shown in (**f**)). The dashed dark red line shows the AIUPred/IUPred3 order vs disorder threshold at 0.5. **h** DynaMine^24^ backbone dynamic prediction profiles for the same sequences that are shown in (**g**). The dashed dark red line indicates the order (values > 0.769) vs. the disorder threshold. The dashed gray lines are at S2 values of 0.69 and 0.8; predicted values below or above these lines indicate flexibility and rigidity, respectively^24^. **i** IUPred3 disorder profiles for the same sequences that are shown in (**g**, **h**).

We asked the question whether circumstantial evidence for the malleability of the α1-helix can be detected at the sequence level and utilized the AIUPred software at first to obtain the disorder propensity of the human heavy chain sequences of the classical and non-classical human MHCI loci (Fig. 5a, d). The prediction of this software is based on a PDB-trained pairwise interaction potential that is then extrapolated to calculate the likelihood of an amino acid at the sequence level to engage in structure-forming interactions in its vicinity^21,22^. Disorder is then annotated to sequences with a low likelihood for structure formation.

As can be seen in Fig. 5a–c, the α1 domain, particularly the region comprising the α1-helix, displays a disorder likelihood for the HLA-A,-B,-C and -G allotypes above 0.5. HLA-E and -F also show increased disorder in that region, albeit less prominently. By contrast, the α2 domain sequence has a lower propensity to be disordered in all HLA families, including the α2-helix that extends from residues 137–175, thus reflecting distinct sequence signatures for the two domains. With the exception of MICB, which displays a trend similar to that of HLA-A, -B and -C, the other non-classical HLA proteins reveal a lower probability of disorder in both the α1 and α2 domains (Fig. 5d). As shown in Fig. 5e, f, this feature is preserved throughout evolution in all peptide-binding MHCI family members. However, it is much less pronounced in many bony fish and chicken alleles, where the α1 domain has a lower predicted disorder probability. Further analysis of the α1 domain reveals that MHCI proteins in pigs, rats and mice contain sequences with a higher propensity for disorder compared to bony fish alleles (Fig. 5f, g, i, Supplementary Figs. 11c, d and 12a, b), while the predictions for non-human primates (NHPs) are similar to the results for HLA-A and -B. In addition to using AIUPred, which capitalizes on deep learning algorithms, we utilized the more conservative IUPred3^23^, which is based on empirical energy estimations and observed similar trends, albeit with less pronounced overall intensity of predicted disorder in the α1-helix (Fig. 5c, i, Supplementary Fig. 11 and 12a, b) and the entire α1 domain.

To independently evaluate disorder propensities, we employed DynaMine^24^, a program that utilizes NMR chemical shift-based order parameter calculations to predict backbone structural order of proteins on a sequence-based level. Values below 0.769 based on the statistical analysis in the original publication^24^ indicate a reasonable classification threshold for order versus disorder, but it is rather qualitative trends that we interpret on the basis of the many different alleles analyzed here. The same trend of order/disorder propensity is seen for DynaMine as with AIUPred and IUPred3 (Fig. 5h and Supplementary Fig. 12a–c), corroborating the distinct features of the α1 and α2 domains, with the only exception of HLA-E, which displays higher order parameters for α1 compared to α2 in DynaMine as well as IUPred3 predictions (Supplementary Fig. 11a–d and Supplementary Fig. 12a–c). CD1 and MR1 show equal stabilities for the two domains in the DynaMine predictions (Fig. 5h and Supplementary Fig. 12c), while for classical and non-classical MHCI most alleles group slightly below the threshold value in the α1-helix. Importantly, there is large allelic variation within individual species (as well as variation of numbers regarding the available sequences), indicating that individual polymorphisms can have a large impact on helical propensities. This is also seen when performing the disorder propensity analysis for the HLA supertypes defined by Harjanto et al.^25^, which can show large variations between individual members of a given family (Supplementary Fig. 13).

## Discussion

An initial and unanticipated finding of this study was that “upper” disulfide bonds (between the α1-helix and Tsn’s editing loop) could be formed between residues of the α1-helix of the MHCI and the editing loop of Tsn that are up to 17 Å apart from each other in previously published structures. While MD simulations indicate that disulfide bond formation is a rare event between these distal residues, the experimental data show that bond formation can be achieved after 24 h. Clearly, this observation is a first indication that the α1-helix or the binding groove as a whole is a dynamic entity. In-depth structural data on the HLA-A2-αY84C/Tsn-K16C variant complex then shows that the observed dynamics mainly stem from a flexibility of the α1-helix that is induced by cleavage of the peptide and dissociation of the C-terminal dipeptide. Analysis of four representative states shows that upper disulfide bond formation is compatible with a fully formed α1-helix, but interestingly in more than half of the particles used for analysis the helix is not resolved in the electron density, indicating a high degree of flexibility/unfolding (Supplementary Figs. 7 and 8). Similarly, peptide is largely dislocalized in more than half of the states, and density can only be assigned to the first two or three N-terminal residues. Notably, in the states where the α1-helix and/or peptide can be largely assigned to the cryoEM density, the B-factors are high, and the resolution is low for these structural elements (Fig. 3 and Supplementary Fig. 7a–h), indicating that dissociation of the C-terminus of the peptide leads to a general destabilization of the helix and increased peptide dynamics. The two events of helical unfolding and peptide dissociation don’t seem to be causally linked since conformations with the α1-helix formed and peptide largely dissociated, or with the helix not formed but the peptide bound, can be observed in the experimental structures.

A conserved feature of all the states described here is the well-resolved interactions of the 2–4 N-terminal residues of the peptide with the α2-domain of the MHCI molecules in the presence of tapasin. The α2-helix is stabilized by the interaction with the chaperone and provides the side chains of αY159 and αY171, in addition to αY7 and αY99 of the β-sheet floor, as an interaction platform for the peptide. The existence of a tyrosine cradle probably reflects the property of tyrosine to engage in hydrogen bonds as well as π-π-, π-anion or π-cation interactions, thus flexibly accommodating different residue types at the peptide's N-terminal positions. The finding that the intermediate discussed in Fig. 4 is formed under equilibrium conditions indicates that it might occur during either peptide association or dissociation with the peptide's C-terminus, either not yet formed or already released. Circumstantial evidence further suggests that the observed intermediate is on-pathway. Firstly, the conformation of the tyrosine side chains seen in our intermediate state 3 is native-like and maintained in either the PLC or other stably tapasin or TAPBPR-bound structures^4,16,17,26–28^. Secondly, contacts between the peptide and the tyrosines are largely present in stably peptide-bound, crystallized MHCI structures^29^. Thirdly, previous kinetic data with the mouse allotype H-2D^b^ were clearly indicative of an early encounter complex that needs to reconfigure during stable MHCI-peptide formation^30^. The authors of that study hypothesized that such a two-step process would allow low-affinity peptides to more easily dissociate and thus introduce a peptide selection step. Our structural model provides evidence for the interactions that are required for such an intermediate to be formed.

**Fig. 6:**
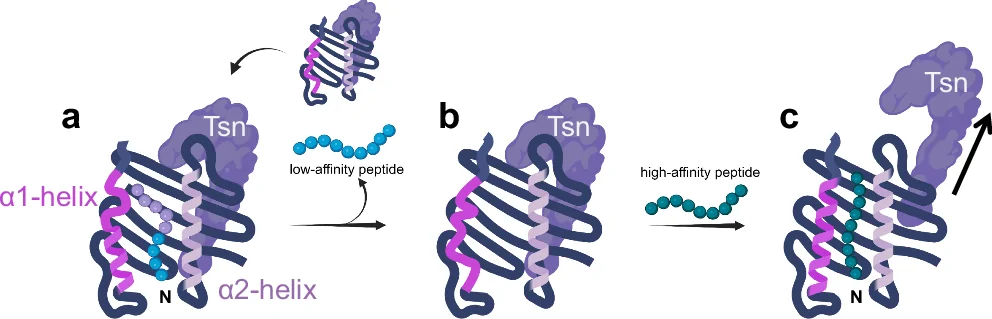
Mechanism of how peptide release and exchange might be coupled to the conformational changes in the MHCI binding groove. Schematic of the role of Tsn in facilitating peptide release and potentially peptide binding. **a** Tsn (violet) stabilizing a partially folded pMHCI complex, where only the first four N-terminal residues (light blue) are stably bound, and the α1-helix (magenta) is only partially folded. The peptide would primarily be coordinated by the tyrosine cradle from the β-sheet floor and the α2-helix (light violet). **b** The competition between Tsn and the C-terminus of the peptide ultimately leads to dissociation, as the peptide lacks sufficient affinity to fully bind. In this state, the α1-helix may be partially folded or in equilibrium between a partially and a mostly unfolded state. **c** A subsequently bound peptide might compete with Tsn more effectively, in which case the α1-helix folds completely and both helices are closing in on the peptide's C-terminus, leading to a weakening of the MHCI-Tsn interface and dissociation of the stable pMHCI complex away from the PLC. Created in BioRender. Mitlehner, A. (2026) https://BioRender.com/y7vsz39^71^.

Thus, a model arises that highlights how helical transitions and peptide binding/dissociation impact on the fate of an MHCI-peptide complex that is entrapped in the PLC. Upon binding of a peptide, several forces are at play: Folding of the α1-helix is unfavorable if the peptide is dissociated or only weakly bound. The folding-unfolding transition of the α1-helix likely slows down initial peptide binding, as it was observed by kinetic measurements^30^. This allows peptides to sample many different conformations more easily and may lead to complexes that are characterized by low *on* rates. α1 helical folding then still occurs with a certain likelihood (state 2), and once it is formed, the critical residue αY84 assumes a position that is compatible with the coordination of the C-terminus of the peptide. However, the Tsn editing loop competes with the space occupied by αY84, constantly “tickling” the F-pocket and thereby sensing the stability of the whole binding groove. In case Tsn interference with the peptide’s C-terminus is effective, the likelihood that the peptide leaves the groove is increasing. In case the peptide C-terminus is stably bound, the helices in the vicinity of the F-Pocket are closing in, the grip of Tsn on the α2-helix is loosened, and the MHCI-peptide complex may leave the PLC as a whole (Fig. 6).

How do our experimental data and scheme relate to other structural studies? In the PLC structure (PDB ID: 7QPD^4^), the α1-helix is fully formed, and no peptide was modeled^4^. B-factors for the MHCI in the PLC are highest for the N-terminal part of the α1-helix, similar to our state 1 and state 2 structures. Notably, a different allotype (HLA-A03) was present in the PLC, and helical stabilities may differ for individual MHCI alleles. In the structure of the HLA-B*44:05 allotype complexed to Tsn, both helices are formed in the presence of a covalently linked 6mer, but density is missing for the peptide^16^. In the TAPBPR-bound HLA-A2, the α1-helix is partially unfolded, and the six N-terminal residues of a peptide can be assigned after photocleavage into a 7mer and a dipeptide (as done in this study)^18^. Thus, we propose that different states along the peptide dissociation pathway have previously been seen, depending on the exact composition of the complexes and the structural analysis method employed. Confirmingly, certain MHCI states in this study such as state 1 resemble the MHCI structure within the PLC or obtained from crystallographic analysis of the Tsn/ERp57/MHCI complex^4,16,17^. Similarly, the structure of HLA-A2 in complex with TAPBPR^18^ shows a good superposition with states 1 and 3 (Fig. 4), indicating that Tsn and TAPBPR converge on stabilizing the α2-helix, but allow for unfolding of the α1-helix in the chaperone-bound form. In contrast, conformational dynamics of an isolated HLA-A2-peptide complex comprises the α2-helix, thereby defining a dynamic state that is recognized by the tapasin homolog TAPBPR, which in turn stabilizes the α2-helix^31^.

What are the implications of this model of tapasin for different allotypes? A primary function of tapasin, according to our results, is to allow stable pMHCI complexes to be formed through the transient but moderately stable intermediate states required for efficient association and dissociation of peptides. Depending on the intrinsic stabilities of the α1- and α2-helices in distinct MHCI variants, the individual allotypes themselves will differ in their likelihood to occupy these states, similar to what has been observed for MHC class II^32–34^.

The disorder propensity analysis performed here (Fig. 5 and Supplementary Fig. 12) might be one measure of these allelic differences, and it will be interesting to see how far helical flexibility modulates the peptide repertoires that are presented by individual allotypes. The propensity for disordered states of the α1-helix seems to have emerged as an evolutionary feature, as bony fish alleles show much lower disorder probabilities compared to species arising later in evolution. This evolutionary shift towards higher disorder propensities of the α1 domain could reflect the mere consequence of polymorphisms, albeit the α2-helix implemented polymorphisms at a similar pace, and the resulting sequences do not show the same probability increase for disorder. Notably, those non-classical HLA molecules that do not present peptides as ligands display α1 domains with low disorder propensity, indicating that α1 helical malleability co-evolved with peptide binding. Interestingly, the peptide-binding non-classical HLA-E and HLA-F MHCI molecules feature α1-helices with lower disorder propensities (Fig. 5a), while the HLA-G α1-helix displays high predicted flexibility. HLA-E molecules bind to a more restricted set of peptides, and their binding groove has been suggested to be open but more rigid compared to classical MHCI^35^. HLA-F molecules are unique in their peptide presentation mode, since the presence of a bulky tryptophan at the beginning of the α1-helix prevents anchorage of the N-terminus and thus allows binding of longer peptides that are mainly anchored by the P9 residue^36^. Thus, flexibility of the binding groove accommodating the P2 residues is not required. HLA-G has been found to present a wide variety of canonical peptides with an anchor residue signature that resembles classical MHCI^37^, suggesting that helical flexibility leads to the presentation of a more extensively edited peptide repertoire compared to its rigid counterparts.

Independent of peptide selection, α1 helical unfolding events might be of relevance for isolated heavy chains that have been observed on the surface of cells. For example, it would be interesting to evaluate whether the stability of the α1-helix relates to the differences in heavy chain clustering at the cell surface observed for different allotypes^38^.

A final reason for the necessity of a rather flexible α1-helix could be that the recycling of suboptimal loaded MHCI proteins, which escaped the PLC without sufficient peptide editing, is conformation-dependent. Namely, the UDP-glucose:glycoprotein glucosyltransferase 1 (UGGT1) is suggested not to recognize peptide-bound MHCI if it is fully folded. Moreover, it was shown that TAPBPR binding can induce unfolding of the C-terminal region of the α1-helix^26^, and we argue that such helical unfolding could serve as a recognition signal for the enzyme UGGT1. UGGT1 senses unstable or empty (p)MHCI complexes when bound to TAPBPR (shown for A*68:02) and reglucosylates the mannose core oligosaccharide attached to αN86 directly at the end of the α1-helix of the MHCI molecule^39^. UGGT1 thus has to capture conformations that are unique to empty or suboptimally loaded and TAPBPR-bound MHCI. We suggest that the α1-helix could be the molecular switch that enables enzyme recognition, and given the constructs made available in our study, the community can now probe whether this important step of ER quality control is indeed modulated by MHCI α1-helical malleability.

## Methods

### Peptides

Peptides used in this study were chemically synthetized (GL Biochem (Shanghai) Ltd. or DG peptides, China). The corresponding sequences were KILGK_FITC_VFTV (FITC-KV9), KILGFVFTV (KV9), and KILGFVFJ*V (pKV9), where J stands for 3-amino-3-(2-nitro) phenyl-propionic acid.

### Recombinant protein expression, refolding, and purification

The pET-3d plasmids containing the sequence for the luminal domain of HLA-A*02:01-K127N (1–276 mature protein, linked to a C-terminal Avi-tag, containing either a Y84C, T73C, or G83C mutation) and β_2_m-T5C (1–100 mature protein) were transformed into *Escherichia coli* BL21(DE3) cells (New England Biolabs, Inc., USA). All point mutations were done by site-directed mutagenesis (see also Table S3 for a comprehensive list of mutations). Chains were expressed individually as inclusion bodies in 2xYT medium (Carl Roth GmbH + Co. KG, Germany) supplemented with 100 µg/mL ampicillin. Induction was done at OD_600_ 0.6–0.8 with 1 mM of IPTG before further incubation overnight at 30 °C.

Inclusion body purification was done as described previously^7^. In brief, cells were pelleted and sonicated in lysis buffer (50 mM Tris, 100 mM NaCl, pH 7.5, 0.5 mM MgCl_2_, 1% TritonX-100 and supplemented with fresh 10 mM DTT and 20 µg/mL DNase I (AppliChem GmbH, Germany)) and subsequently pelleted at 15,000 g for 20 min. The supernatant is discarded, and the pellet is washed 4 times in washing buffer (50 mM Tris, 100 mM NaCl, pH 7.5, 0.5 mM EDTA, 0.5% TritonX-100 and supplemented with fresh 10 mM DTT) using sonication. Purified inclusion bodies are resolubilized in (20 mM Tris, pH 8, 0.5 mM EDTA, 4 M urea and 10 mM DTT). Refolding of peptide-loaded MHCI complexes was performed by adding urea-solubilized β_2_m to the refolding buffer (0.4 M arginine, 0.1 M Tris, pH 8.0, 5 mM GSH, 0.5 mM GSSG, 2 mM EDTA) in a dropwise manner at 4 °C. After a one-hour incubation, the peptide and solubilized heavy chain were added, and the refolding was left stirring for 4–5 days before purification. Purification was done by size exclusion chromatography (Superdex200 Increase 10/300 GL, Cytiva, USA) in PBS buffer pH 7.4. Fractions containing the refolded protein were pooled, concentrated, shock frozen in liquid nitrogen and stored at −80 °C.

Human Tsn and the respective mutants (G304C, G304C/K16C, G304C/D12C, G304C/L18C) were co-expressed with ERp57-C60A in Sf9 insect cells using the pFastBac-Dual vector. The construct was available from previous work: Tsn (1-398) with a C-terminal His_6_-tag inserted between *Bam*HI/*Xba*I (New England Biolabs, Inc., USA) and ERp57-C60A (1–504) inserted between *Sma*I/*Sph*I (New England Biolabs, Inc., USA). Cysteine mutants were produced by site-directed mutagenesis. Constructs with Tsn and ERp57 were transfected into Sf9 (*Spodoptera frugiperda*) insect cells using Cellfectin II reagent (Bac-to-Bac® Baculovirus Expression System, Thermo Fisher), following the manufacturer’s instructions.

Cells were harvested 4 days after transfection/infection, and protein purification was done as previously described^7^ by nickel-ion affinity chromatography using Ni-NTA agarose beads (Macherey-Nagel™, Germany) and SEC (Superdex200 Increase 10/300 GL, Cytiva, USA) with PBS pH 7.4 as running buffer. Fractions containing ERp57-Tsn complexes were pooled, concentrated and shock frozen before being stored at −80 °C.

### Thermofluor assay

The influence on the thermal stability of proteins under different buffer conditions was measured with the Mx3005P qPCR system (Agilent, USA) in a 96-well plate format using a laboratory-made screen. Each well contained 135 µl buffer, 10 µl protein (0.1–0.2 µg/µl), and 1 µl of 10x SYPRO Orange dye (Invitrogen, Thermo Fisher Scientific, USA). The three-step program included a pre-incubation for 1 min at 20 °C, and subsequently the temperature was increased by 1 °C within 20 s. The final incubation was performed at 95 °C for 1 min. The data was acquired with MxPro QPCR software (Agilent) and analysed with OriginPro 2023b.

Thermal stability of proteins and the influence of the respective mutations were measured with the StepOne Real-Time PCR system (Applied Biosystems, Thermo Fisher Scientific, USA) in a 96-well plate format. Proteins were diluted to 0.3 mg/mL, and 20 µL protein solution was added per in-plate replicate. Each well was supplied with 5 µL of 25x SYPRO Orange dye (Invitrogen, Thermo Fisher Scientific, USA). Melting temperatures were measured in triplicate using a similar protocol mentioned above, but starting at 25 °C. Data was acquired with the StepOne Software (Thermo Fisher Scientific, USA) and evaluated using OriginPro 2023b.

### Fluorescence polarization

Fluorescence polarization was used to measure peptide exchange rates for pMHCI complexes preloaded with pKV9 peptide. The peptide was not cleaved before measuring. For the experiment 10 µL of 1.2 µM pMHCI was added with or without 10 µL of 1.2 µM Tsn-ERp57 and 10 µL 400 nM of FITC-KV9 into 384-well plates (Corning® 384-well Low Flange Black Flat Bottom Polystyrene NBS Microplate 3575, USA). Additionally, 10 µL of PBS buffer was added to the well if Tsn was used and 20 µL in the wells without Tsn. Association of the fluorescently labeled peptide was monitored for 4–5 h at 25 °C, and values were recorded approximately every 60 s. Excitation and Emission values used to monitor fluorescence polarization were 485 nm and 535 nm (Bandwidth 20 nm), respectively. 400 nM of pure FITC-KV9 peptide were used as a blank, and values were subtracted from the experimental data. All measurements had three technical, in-plate replicates, and three individual experiments were used for averages of apparent *k*_on_ values. Data was recorded on a Spark® multimode microplate reader (Tecan Group Ltd., Switzerland). Analysis and data fitting were done in OriginPro 2023b using the equation: y = y0 – Ae^(-kt)^ with y0 as the maximum value/plateau, A as the amplitude and k as the rate constant. Calculated rates are apparent *k*_on_ values as the MHCI protein is already loaded with peptide.

### Complexformation and gel verification by LC-ESI MS

For disulfide-bridge formation experiments 10 µL of 4 µM photocleaved pMHCI cysteine variant (Y84C, T73C, or G83C) was mixed with 10 µL of 1.5 µM Tsn variant (K16C, D12C, or L18C)-ERp57 complex, and 2 µL of redox buffer was added (5 mM GSH, 50 mM GSSG in PBS pH 7.4). Photocleavage of pKV9-loaded MHCI protein was done by exposing the protein to UV light for 5 min on ice.

Samples for complex formation were incubated overnight at 4 °C before 20 µL were mixed with non-reducing SDS-loading dye and without boiling loaded onto SurePAGE™, Bis-Tris 4–12% gradient SDS-Gel (GenScript Biotech, USA). Gel ran at 140 V for 70 min, was stained with Coomassie Brilliant Blue R-250 and destained with 10% acetic acid.

Bands of interest were cut and processed following a standard protocol for tryptic in-gel digest^40^. In brief, bands were destained, and cysteine residues were reduced and carbamidomethylated to help detect the peptides after trypsin digestion.

Peptides were reconstituted in 10 µL of 0.05% trifluoroacetic acid (TFA) containing 4% acetonitrile, and 1 µL were injected for analysis using an Ultimate 3000 reverse-phase capillary nano-liquid chromatography system coupled to a Q Exactive HF mass spectrometer (Thermo Fisher Scientific, USA). Samples were first loaded onto a trap column (Acclaim PepMap 100 C18, 3 µm, 100 Å, 75 µm i.d. × 2 cm, Thermo Fisher Scientific, USA). After switching the trap column inline, chromatographic separation was performed on an analytical column (Acclaim PepMap 100 C18, 2 µm, 100 Å, 75 µm i.d. × 50 cm, Thermo Fisher Scientific, USA) at a flow rate of 300 nL/min and a column temperature of 55 °C. Mobile phase A consisted of 0.1% formic acid in water, and mobile phase B of 0.1% formic acid in 80% acetonitrile/20% water. The column was pre-equilibrated with 5% B, and peptides were separated using a linear gradient from 5% to 44% B over 35 min. The mass spectrometer was operated in data-dependent acquisition mode. Survey MS scans were acquired in the range of m/z 350–1650 at a resolution of 60,000, followed by MS/MS scans of the 15 most intense precursor ions at a resolution of 15,000. Dynamic exclusion was set to 20 s. Automatic gain control (AGC) targets were 3 × 10^6 for MS scans and 1 × 10^5^ for MS/MS scans, with maximum injection times of 20 ms for MS and 25 ms for MS/MS. The isolation window was set to 1.4 m/z, and higher-energy collisional dissociation (HCD) was performed with a normalized collision energy of 27. Charge states that were unassigned, singly charged, or higher than 6+ were excluded from fragmentation.

Data processing and peptide identification were carried out using the Mascot software suite (Mascot Server v2.7, Mascot Daemon v2.7; Matrix Science). Spectra were searched against the UniProt human reference proteome supplemented with a custom database containing the expressed proteins and their respective mutations. Enzyme specificity was set to trypsin, allowing up to two missed cleavages. Precursor and fragment ion mass tolerances were set to 15 ppm and 0.02 Da, respectively. Carbamidomethylation of cysteine was specified as a fixed modification, whereas methionine oxidation and protein N-terminal acetylation were set as variable modifications. Peptides were considered confidently identified when their Mascot scores exceeded the homology threshold, with a significance level of *P* < 0.05 determined by decoy database searches.

### CryoEM sample preparation and data acquisition

For cryoEM sample preparation 450 µL of 4 µM pMHCI (A*02:01-K127N/Y84C//β_2_m-T5C//pKV9) were photocleaved as described above, mixed with 450 µL of 1.5 µM Tsn-G304C/K16C//ERp57-C60A complex and 100 µL of redox buffer was added before the complex was incubated overnight at 4 °C.

Complex was purified by size-exclusion chromatography (Superdex200 Increase 10/300 GL, Cytiva, USA) and concentrated to 5 mg/ml in SEC buffer (20 mM HEPES, 100 mM NaCl, pH 7.5) for grid preparation. Quantifoil R1.2/1.3 holey carbon grids (Großlöbichau, Germany) were glow-discharged (10 mA, 60 s), and 3.8 µl of the sample supplemented with a final concentration of 0.15% n-octylglucoside was applied. Vitrification was performed using a Vitrobot Mark IV (ThermoFisher Scientific; blot time: 4 s, wait time: 4 s, blot force: -10, 100% humidity, 10 °C), followed by plunging into liquid ethane.

Data was acquired on a FEI Titan Krios G3i transmission electron microscope (FEI Company, Eindhoven, Netherlands) operated at 300 kV equipped with a Falcon 3EC detector. Movies were recorded for 40.57 s in counting mode, accumulating a total electron flux of ~42 el/Å^2^ distributed over 33 fractions. The nominal magnification was set to 96,000x, corresponding to a calibrated pixel size of 0.819 Å/px. Automated data acquisition was conducted with EPU3.8 (Thermo Fisher Scientific; Table S2).

### CryoEM data analysis

All image analysis steps were done with cryoSPARC (version 4.5.0)^41^. Movie alignment was done with patch motion correction, generating Fourier-cropped micrographs (pixel size 1.638 Å/px), and CTF estimation was conducted by Patch CTF. Class averages of manually selected particle images were used to generate an initial template for reference-based particle picking from 5699 micrographs. 2,281,616 particle images were extracted with a box size of 144 px and Fourier-cropped to 72 px for initial analysis. Reference-free 2D classification was used to select 65,616 particle images for ab initio reconstruction to generate an initial 3D reference for subsequent classification by heterogeneous 3D refinement of the entire data set. A final subset of 770,681 particle images was subjected to local motion correction and uncropped extraction with 288 px box size. Non-uniform (NU) refinement after CTF correction gave a consensus reconstruction at 3.1 Å resolution, locally extending down to 2.4 Å.

Due to structural flexibility in the peptide binding pocket, 3D variability analysis was conducted to further isolate four distinct conformational sub-states, which were individually refined by NU refinement to global resolutions between 3.5 Å and 3.6 Å.

### Model building, refinement and analysis

The initial model of the peptide loading module was assembled by placing the respective high-resolution crystal structures into the cryoEM reconstruction: heavy chain and β_2_m (PDB ID: 6O9B)^42^ Tsn and ERp57 (PDB ID: 3F8U)^43^. Single proteins and their respective domains were adjusted by rigid-body fitting. Missing loop regions were manually built. Glycosylation of Tsn was added in COOT^44^. The structures were refined by segmental real-space refinement using Coot (version 1.1.13)^45^. All structures were refined by iterative rounds of real-space refinement in PHENIX (version 1.21.2_5419)^46^ and manual adjustment in Coot. The structural models were evaluated with MolProbity (version 4.5.2)^47^. Structure figures were prepared with PyMOL (version 3.1.2, Schrödinger) and ChimeraX^48^.

### Molecular dynamics simulations

We performed simulations of the HLA-A*02:01-Tsn complex to compare to experiments. To assemble a complex with the HLA-A*02:01 allotype along with Tsn, we first took the structure of the MHCI heavy chain, microglobulin and peptide from an existing structure for such allotype (PDB ID: 1I1F), performed a structural alignment to a structure of the whole PLC with allotype HLA-A*03:01 (PDB ID: 7QPD) and then retained only the chains for HLA-A*02:01 and Tsn. For the mutant simulation of Fig. 1d, e, we further exclude the peptide from the assembled complex, as we desired to test only if the S–S bonds were possible to form. Once constructed, the system was processed in Charmm-GUI^49^ first to perform the wanted mutations and then to reconstruct the missing atoms from side chain systems were posteriorly solvated in a rectangular water box with a 12 Å padding on every dimension. Na, Cl ions and hydrogens were added to achieve a salt concentration of 0.15 µM and a pH of 7.4. The PROTOSS server was used to predict the protonation states of histidines^50^.

Because our aim is solely to probe conformational plasticity rather than explicit disulfide-bond formation, a conventional molecular dynamics simulation with a state-of-the-art classical force field provides an adequate level of description^51^. Therefore, for our simulations, we use the Amberff14SB force field with TIP3P water. A non-bonded interaction cutoff of 0.9 nm and a switch distance of 7.5 Å for the Lennard-Jones interactions were applied, along with constraints for the hydrogen bond lengths of the system. The long-range electrostatic forces were calculated using Particle Mesh Ewald. We use Langevin dynamics at a temperature 300 K with a friction coefficient of 0.1 ps^−1^ and a timestep of 2 fs.

For relaxing the solvent, the simulation protocol started with a multi-step equilibration with a constraint over the Cα and Cβ positions of the protein. The equilibration steps consisted on:

1. Energy minimization
2. Constant pressure and temperature (NPT) simulation via a Monte Carlo Barostat at 1 atm with a temperature ramp from 5 to 300 K in 5 K intervals steps while running 2 ps per temperature.
3. 200 ps of NPT simulation at 300 K at 1 atm.
4. 200 ps of constant volume and temperature (NVT) simulation at 300 K.

After this, production runs were done using an NVT ensemble. For the 9 variants (MHCI T73C, G83C, Y84C; Tsn D12C, K16C, L18C), we ran 16 independent trajectories of 1 µs each, amounting to a total of ~144 µs.

For the Free Energy Surface (FES) generation of the Y84C-K16C variant (Supplementary Fig. 9), an adaptive sampling strategy was followed^52^. We generated three generations of trajectories, the first one starting from the CryoEM structures, and subsequent ones starting from simulation frames from existing simulations. These simulations also included an explicit constraint of the S–S disulfide bond of the mutated residues. 115 trajectories of ~1 µs were obtained, for a total of ~115 µs of aggregated time. All the simulations were performed using Python and the OpenMM MD engine (version 8.0.0)^53^. Trajectory processing was done via the MDTraj package (version 1.11.0)^54^. Markov State Model reweighting and the Hidden Markov Model estimation were done using the Deeptime package (version 0.4.5)^55^.

The timescales associated with conformational changes in MHC are typically in the order of hundreds of microseconds^32,33^, which is covered by our trajectories. To assess the convergence of the simulations, for the mutational analysis of the 9 variants, we visualize the timeseries of the S-S distances shown in Fig. 2 and Supplementary Fig. 3 (Supplementary Fig. 14). For the FES of the Y84C-K16C variant, we review the implied timescales associated with the Markov-State Model (Supplementary Fig. 15).

### Disorder predictions with AIUPred, IUPred3 and DynaMine

For all three prediction tools, the sequences were curated in the same way to improve comparability. With the exception of the sequences for CD1, MR1 and mouse MHCI proteins, all sequences were obtained from the IPD-IMGT/HLA and IPD-MHC Database^56–58^ (retrieval in Oct. 2025). MR1 (retrieval Feb. 2026), CD1 and mouse sequences were obtained from uniprot^59^. In the case of MR1, all sequence variants were analysed. All sequences were trimmed in order to remove the signal sequence and regions after the α3 domain and sorted into classical and non-classical alleles; null alleles were excluded from analyses. Sequences were aligned using Kalign 3^60^. Curated and aligned fasta files were used for the prediction tools AIUPredv2^22^, IUPred3^23,24^ and DynaMine^24^. All predictions were performed and visualized with Python (version 3.12.2) using the packages Pytorch^61^ (version 2.0.1), NumPy^62^ (version 1.26.4), SciPy^63^ (version 1.13.0), biopython^64^ (version 1.86) and DynaMine as a part of the b2bTools^65^ (version 3.0.7). Additionally, the packages pandas^66^ (version 2.3.3), seaborn^67^ (version 0.13.2) and matplotlib^68^ (version 3.10.8) were used especially for visualization. Code was developed with assistance from GitHub Copilot (GitHub, Inc.)^69^, an AI-powered coding assistant based on large language models (in this case, Claude Sonnet 4.6 and Gemini 3 were used as agents), used for code generation and autocompletion.

### Reporting summary

Further information on research design is available in the Nature Portfolio Reporting Summary linked to this article.

## Supplementary information

Supplementary Information

Transparent Peer Review file

Reporting Summary

Description of Additional Supplementary Files

Supplementary Movie 1

## Source data

Source Data

## Data Availability

Disorder predictions generated in this study have been deposited in the Figshare database under [https://figshare.com/s/89981d2d3e070dba36c1]. The trajectories for the molecular dynamics studies can be found in Zenodo at https://doi.org/10.5281/zenodo.21915336 and https://doi.org/10.5281/zenodo.21898862. The cryo-EM maps developed in this study have been deposited in the Electron Microscopy Data Bank (EMDB) under accession codes EMD-55112 [https://www.ebi.ac.uk/emdb/EMD-55112 (State 1), EMD-55113 (State 2), EMD-55114 (State 3) and EMD-55111 (State 4). Atomic coordinates have been deposited to the Protein Data Bank (PDB) under accession codes 9SQN (State 1), 9SQO (State 2), 9SQP (State 3) and 9SQM https://doi.org/10.2210/pdb9SQM/pdb (State 4). Source data are provided with this paper.
