# Supplementary material for: Dynamic states of an MHC class I molecule during peptide exchange
Source: Nat Commun. 2026 Sep 10;17:9697. doi: 10.1038/s41467-026-77731-6 (PMC13562589; doi:10.1038/s41467-026-77731-6)
Supplement: Supplementary file 1 [file 41467_2026_77731_MOESM1_ESM.pdf]

Supplementary Information

Dynamic states of an MHC class I molecule during peptide exchange

Alexandra Mitlehner[1], Aldo S. Pasos-Trejo[2], Moritz Becker[1], Huan Lan[1], Lennard Berg[1], Tarek Hilal[3,4,5], Bernhard Loll[4], Benno Kuropka[1,5], Cecilia Clementi[2] & Christian Freund[1]. Freie Universität Berlin, Germany.

1 Laboratory of Protein Biochemistry, Institute of Chemistry and Biochemistry, Freie Universität Berlin, 14195 Berlin, Germany.

2 Department of Physics, Freie Universität Berlin, Arnimallee 12, 14195 Berlin, Germany

3 Research Center of Electron Microscopy, Institute of Chemistry and Biochemistry, Freie Universität Berlin, 14195 Berlin, Germany.

4 Laboratory of Structural Biochemistry, Institute of Chemistry and Biochemistry, Freie Universität Berlin, 14195 Berlin, Germany.

5 Core Facility BioSupraMol, Freie Universität Berlin, 14195 Berlin, Germany

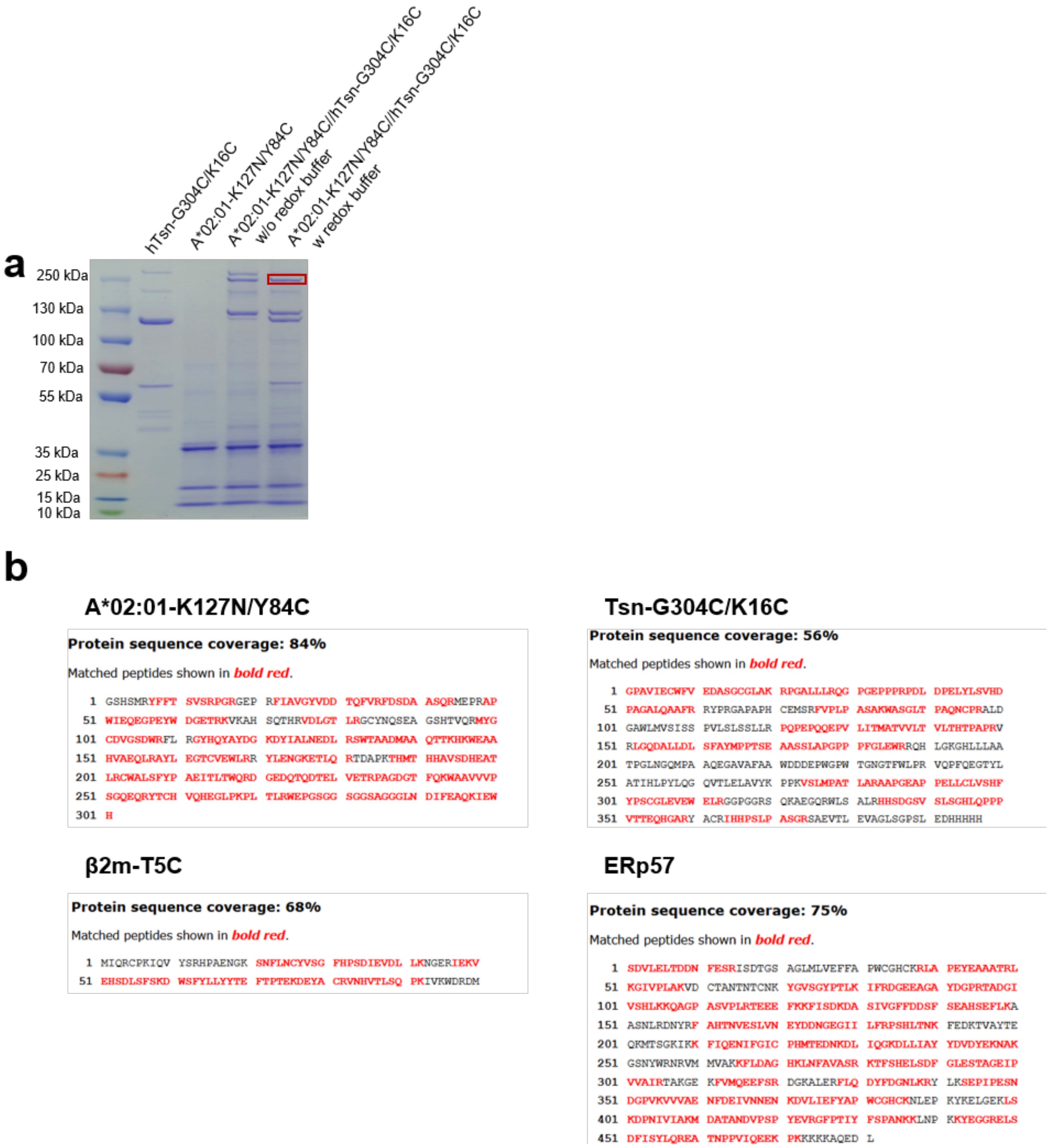

Supplementary Fig. 1: Mass spectrometric characterization of the MHCI-Tsn-Erp57 complex. **a)** SDS-gel of the complex of A*02:01-K127N/Y84C with hTsn-K16C. The indicated band was used for MS verification. **b)** Sequence coverage of proteins unambiguously identified by LC-MS/MS analysis after in-gel digestion. Identified peptides are highlighted in red.

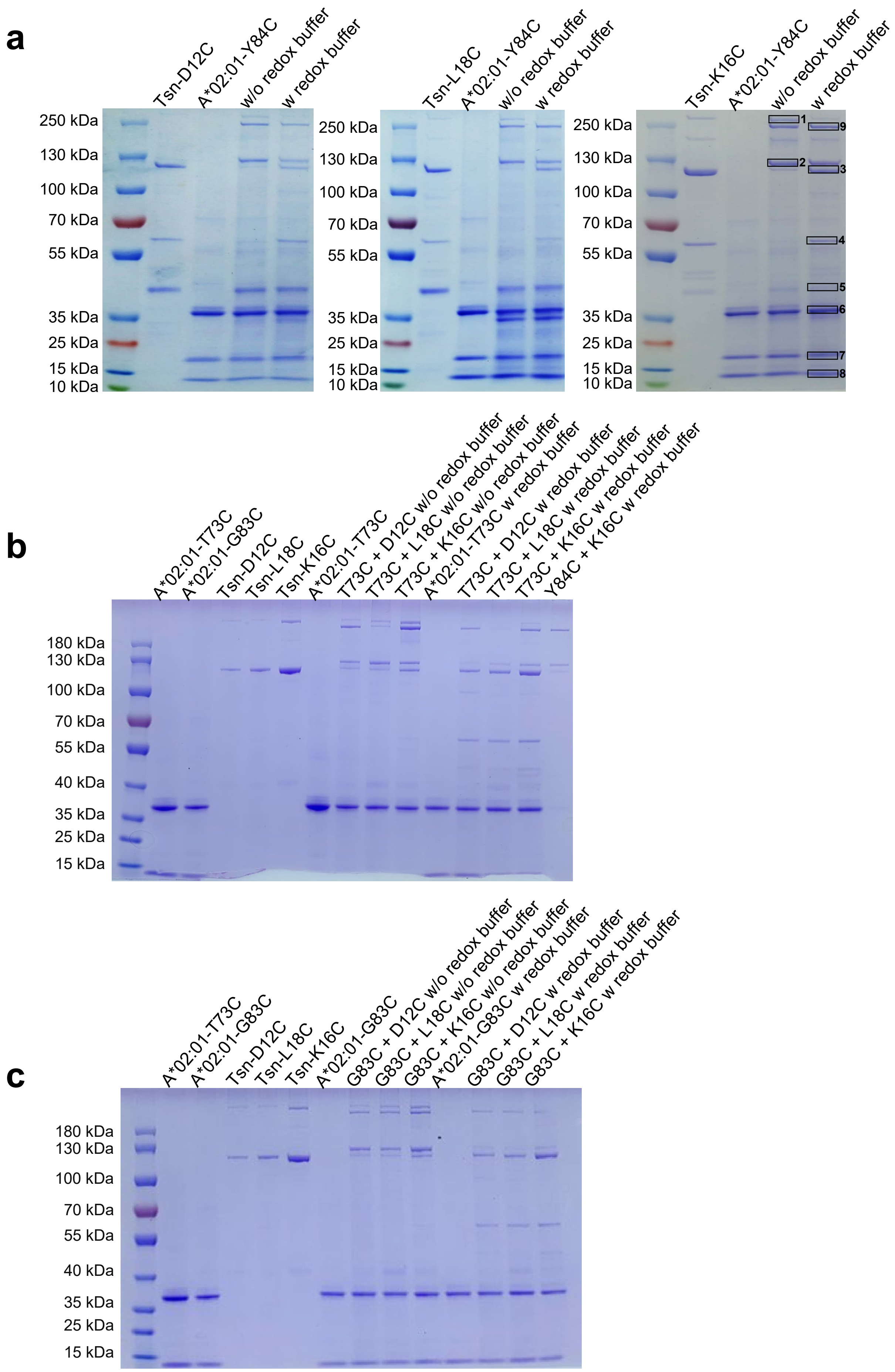
a
Tsn-D12C
A*02:01-Y84C
w/o redox buffer
w redox buffer
250 kDa
130 kDa
100 kDa
70 kDa
55 kDa
35 kDa
25 kDa
15 kDa
10 kDa
Tsn-L18C
A*02:01-Y84C
w/o redox buffer
w redox buffer
Tsn-K16C
A*02:01-Y84C
w/o redox buffer
w redox buffer
1
2
3
4
5
6
7
8
9
b
A*02:01-T73C
A*02:01-G83C
Tsn-D12C
Tsn-L18C
Tsn-K16C
A*02:01-T73C
T73C + D12C w/o redox buffer
T73C + L18C w/o redox buffer
T73C + K16C w/o redox buffer
A*02:01-T73C w redox buffer
T73C + D12C w redox buffer
T73C + L18C w redox buffer
T73C + K16C w redox buffer
Y84C + K16C w redox buffer
180 kDa
130 kDa
100 kDa
70 kDa
55 kDa
40 kDa
35 kDa
25 kDa
15 kDa
c
A*02:01-T73C
A*02:01-G83C
Tsn-D12C
Tsn-L18C
Tsn-K16C
A*02:01-G83C
G83C + D12C w/o redox buffer
G83C + L18C w/o redox buffer
G83C + K16C w/o redox buffer
A*02:01-G83C w redox buffer
G83C + D12C w redox buffer
G83C + L18C w redox buffer
G83C + K16C w redox buffer
180 kDa
130 kDa
100 kDa
70 kDa
55 kDa
40 kDa
35 kDa
25 kDa
15 kDa

Supplementary Fig.2: Non-reducing SDS-PAGEs showing complex formation. Gels used were 4-12% Bis-Tris SurePAGE gels. Gels were run at 140 V for 70-75 min. Ladder used in A is PageRuler™Plus from Thermo Fisher Scietific. For B and C PageRuler™ from Thermo Fisher Scientific was used. **a)** Complexformation SDS-PAGEs for A*02:01-K127N/Y84C//β2m-T5C//pKV9 with ERp57-C60A//tapasin-G304C/D12C, ERp57-C60A//tapasin-G304C/L18C and ERp57-C60A//tapasin-G304C/K16C. Annotated bands are: 1-Tsn/ERp57 multimer, 2-Tsn/ERp57-β2m, 3-Tsn/ERp57, 4- ERp57, 5-Tsn, 6-A2-heavy chain, 7-β2m dimer, 8-β2m, 9- disulfide-bridged A2-αY84C/Tsn-K16C complex. **b)** Complexformation SDS-PAGEs for A*02:01-K127N/T73C//β2m-T5C//pKV9 with ERp57-C60A//tapasin-G304C/D12C, ERp57-C60A//tapasin-G304C/L18C and ERp57-C60A//tapasin-G304C/K16C. **c)** Complexformation SDS-PAGEs for A*02:01-K127N/G83C//β2m-T5C//pKV9 with ERp57-C60A//tapasin-G304C/D12C, ERp57-C60A//tapasin-G304C/L18C and ERp57-C60A//tapasin-G304C/K16C.

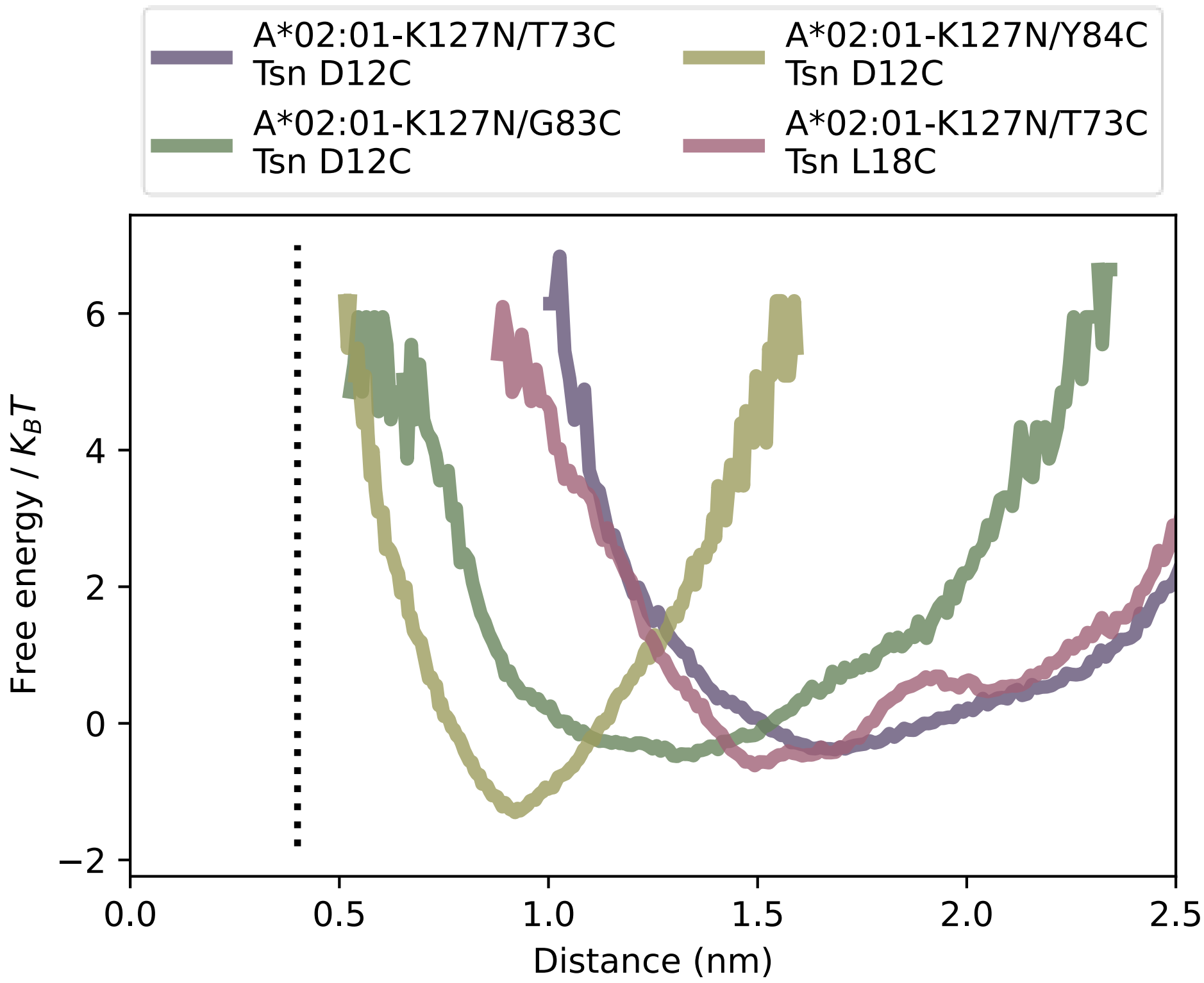

Supplementary Figure 3: Free energy profiles for the S-S distances from MD simulations starting without a formed disulfide bridge as starting point of other variants which are unable to sample comformation below the threshold of 4 Å.

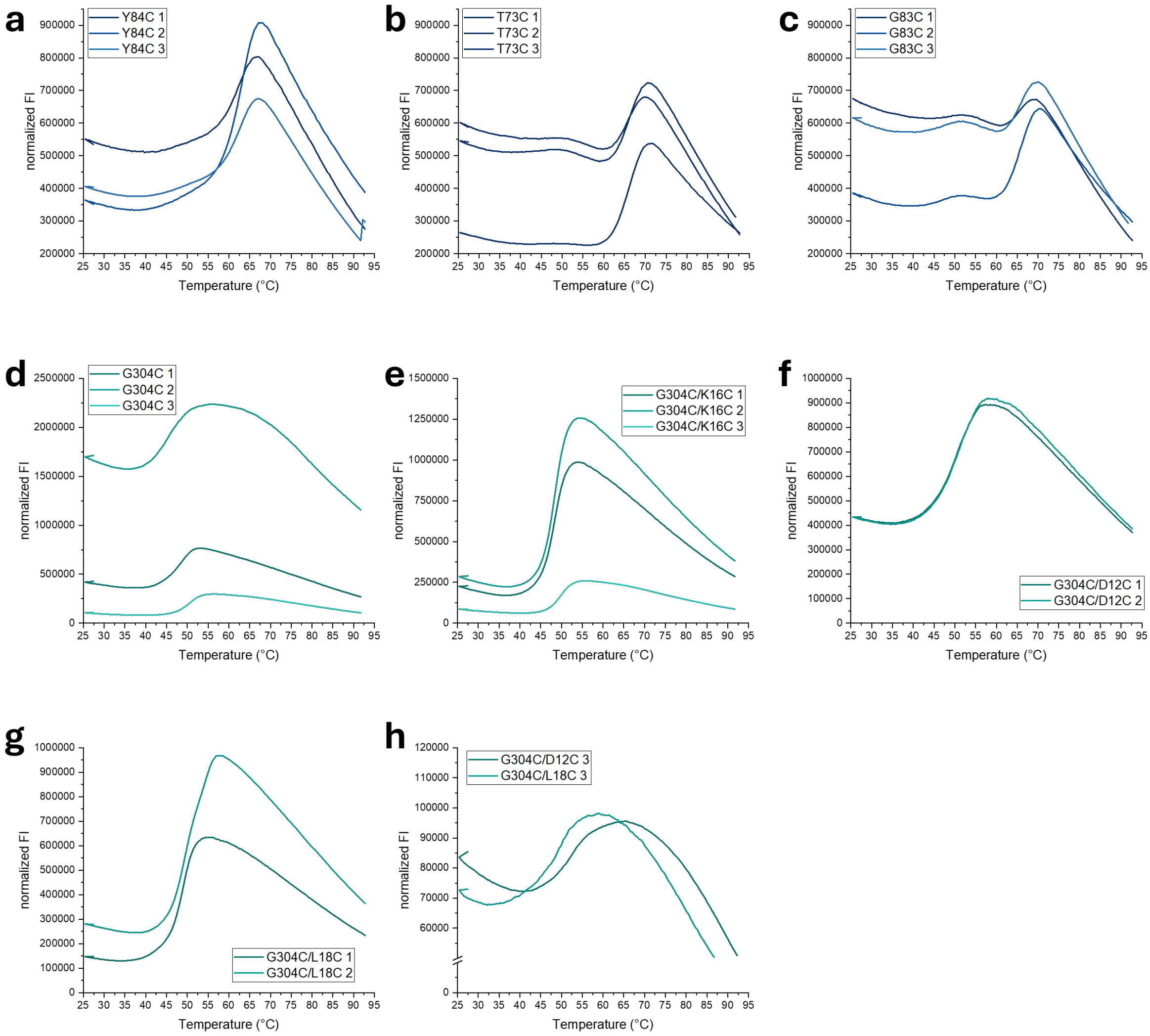

Supplementary Fig. 4: Melting curves in replicates for **a)** A*02:01-K127N/Y84C, **b)** A*02:01-K127N/T73C, **c)** A*02:01-K127N/G83C, **d)** Tsn-G304C//ERp57-C60A, **e)** Tsn-G304C-K16C//ERp57-C60A, **f)** Tsn-G304C-D12C//ERp57-C60A, **g)** Tsn-G304C-L18C//ERp57-C60A and **h)** third replicate for Tsn-G304C-D12C//ERp57-C60A and Tsn-G304C-L18C//ERp57-C60A .

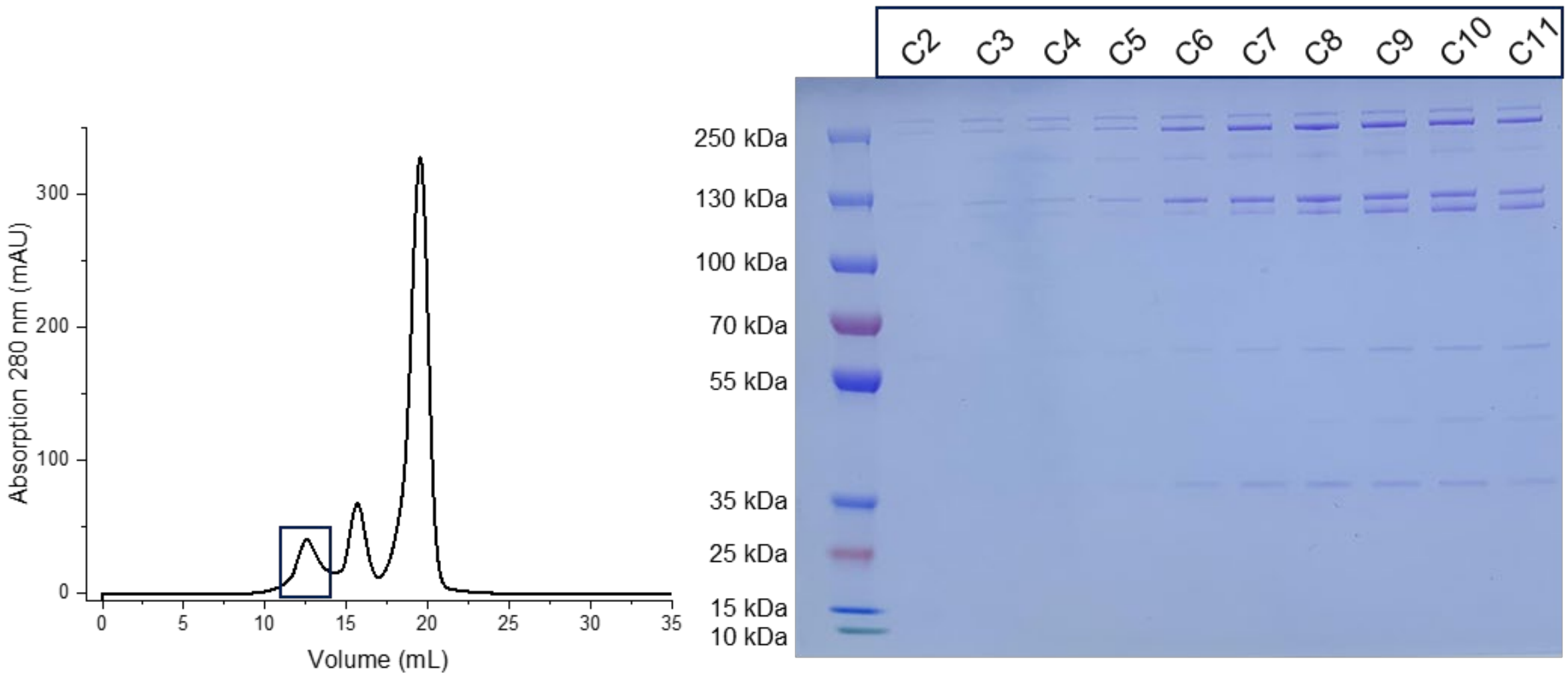

Supplementary Fig. 5: Chromatogram and non-reducing SDS-PAGE for A*02:01-K127N/αY84C//β_2m-T5C//pKV9//ERp57-C60A//Tsn-G304C/K16C complex purification. Indicated fractions were submitted to non-reducing SDS-PAGE (4-12% Bis-tris, 140 V, 75 min).

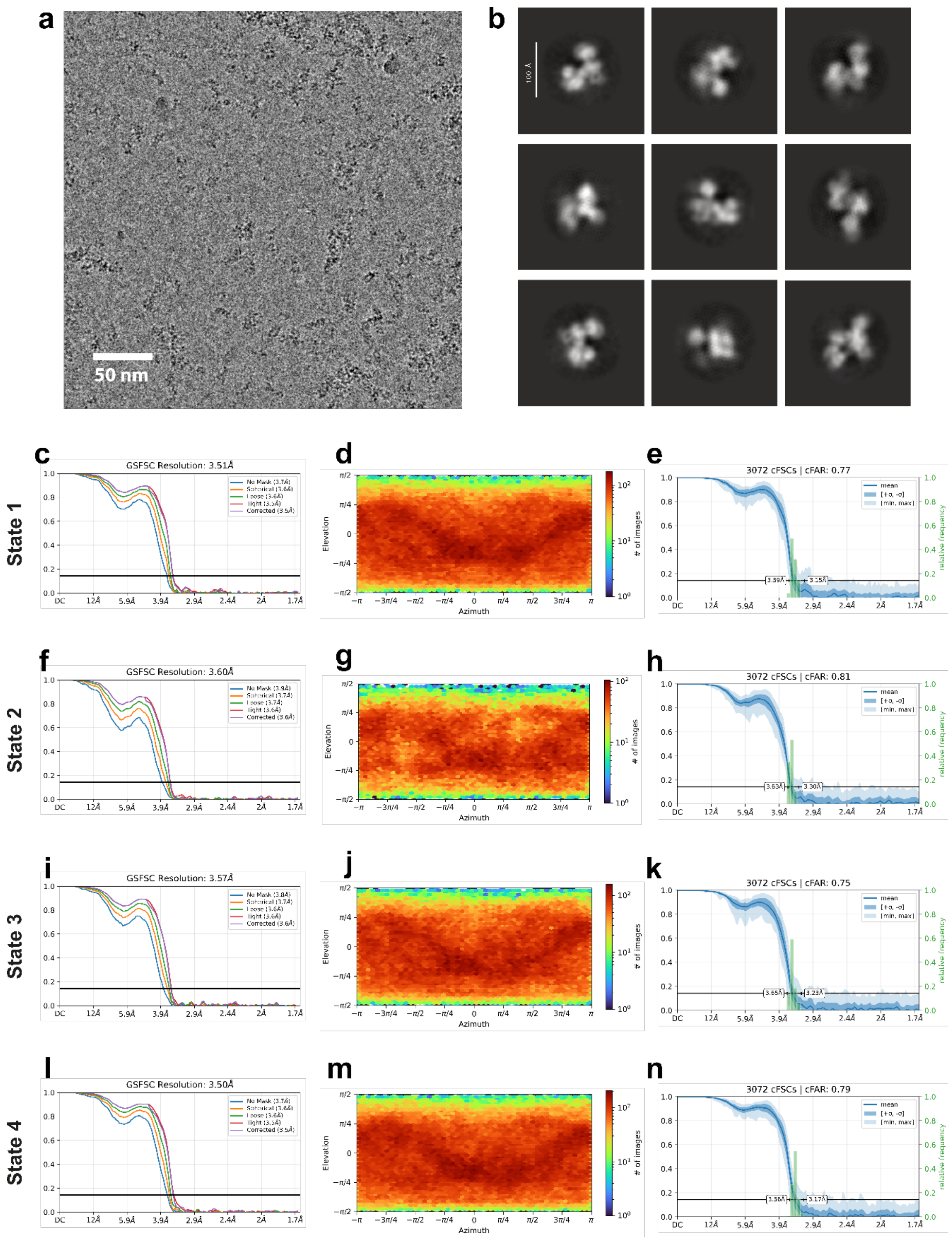

Supplementary Fig. 6: CryoEM analysis of the MHCI-Tsn-Erp57 complex. **a)** representative cryoEM micrograph. **b)** Selected 2D class averages. Qualitative metrics of the 3D reconstruction for the indicated state 1 - 4 **c, f, i, l**) Gold-standard fourier shell correlation curve obtained during NU refinement. **d, g, j, m**) Viewing angle distribution as obtained during Non-Uniform (NU) refinement. **e, h, k, n)** cFSC analysis indicates good projection angle coverage for all reconstructions.

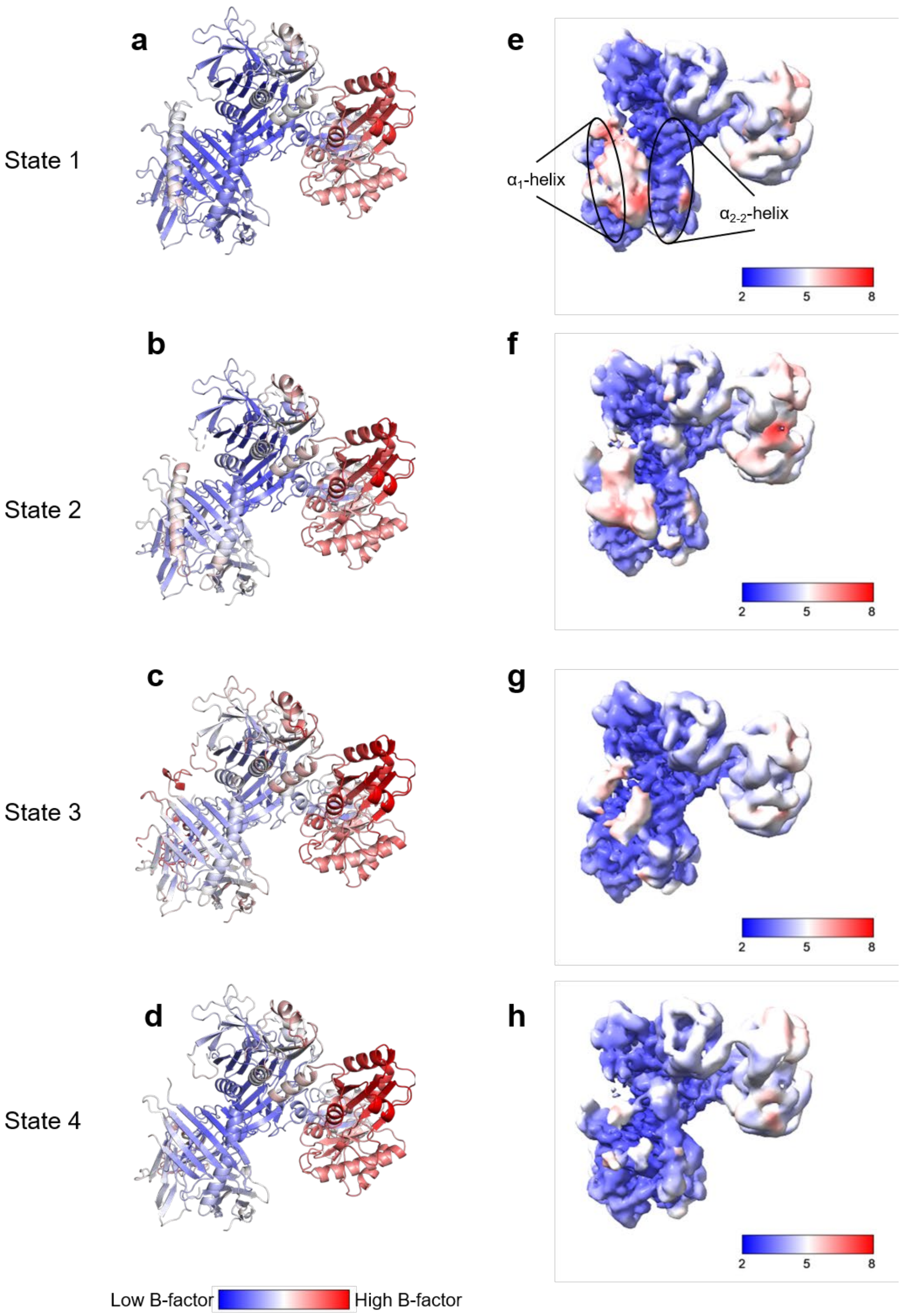

Supplement Fig. 7: B-factor and local resolution of all four states of our cryoEM model and electron densities. Range of resolution is given in Å. Close up of the structures shows the peptide binding groove. **a)** and **e)** show state 1, **b)** and **f)** show state 2, **c)** and **g)** show state 3 and **d)** and **h)** show state 4. B-factor images were created with PyMOL[1] and local resolution mapping visualized using ChimeraX1.10[2].

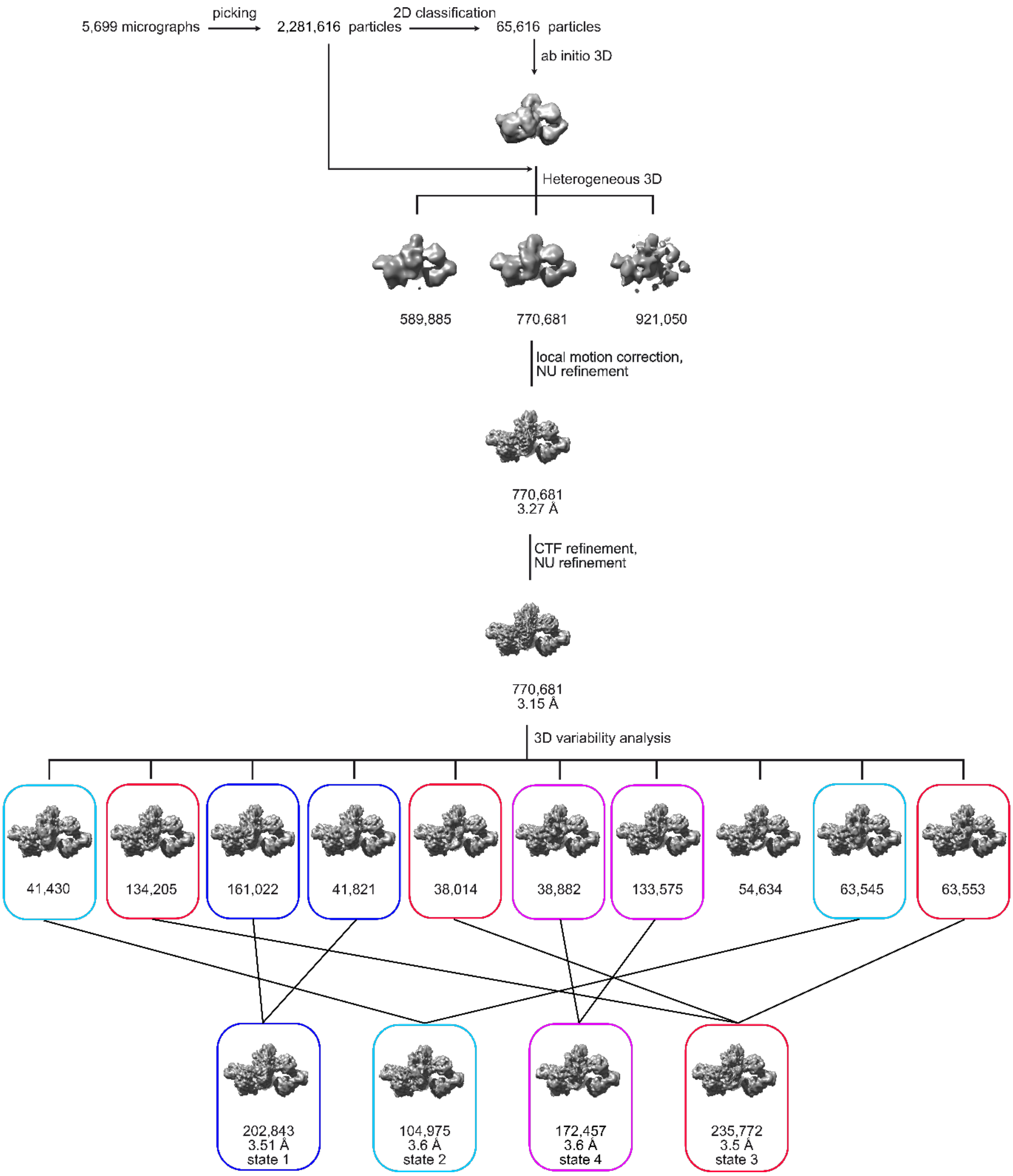

Supplementary Fig. 8: CryoEM data analysis workflow. A total of 2,281,616 particles were picked from 5,699 micrographs and subjected to reference-free 2D classification. 65,616 particles were selected for ab initio 3D reconstruction which resulted in a reasonable representation of the MHCI-Tsn-Erp57 complex. This was used as reference for heterogeneous refinement of the entire dataset after which 770,681 particle images were selected for further analysis. Local motion correction, followed by NU refinement gave a consensus reconstruction at 3.27 Å resolution that could be improved to 3.15 Å by CTF refinement. Guided by less defined density within the peptide binding pocket, we applied 3D variability analysis to further dissect heterogeneity within the dataset into a total of 10 classes. Similar appearing structures were combined after careful inspection as indicated and refined individually into states 1-4 at global resolutions between 3.5 Å and 3.6 Å.

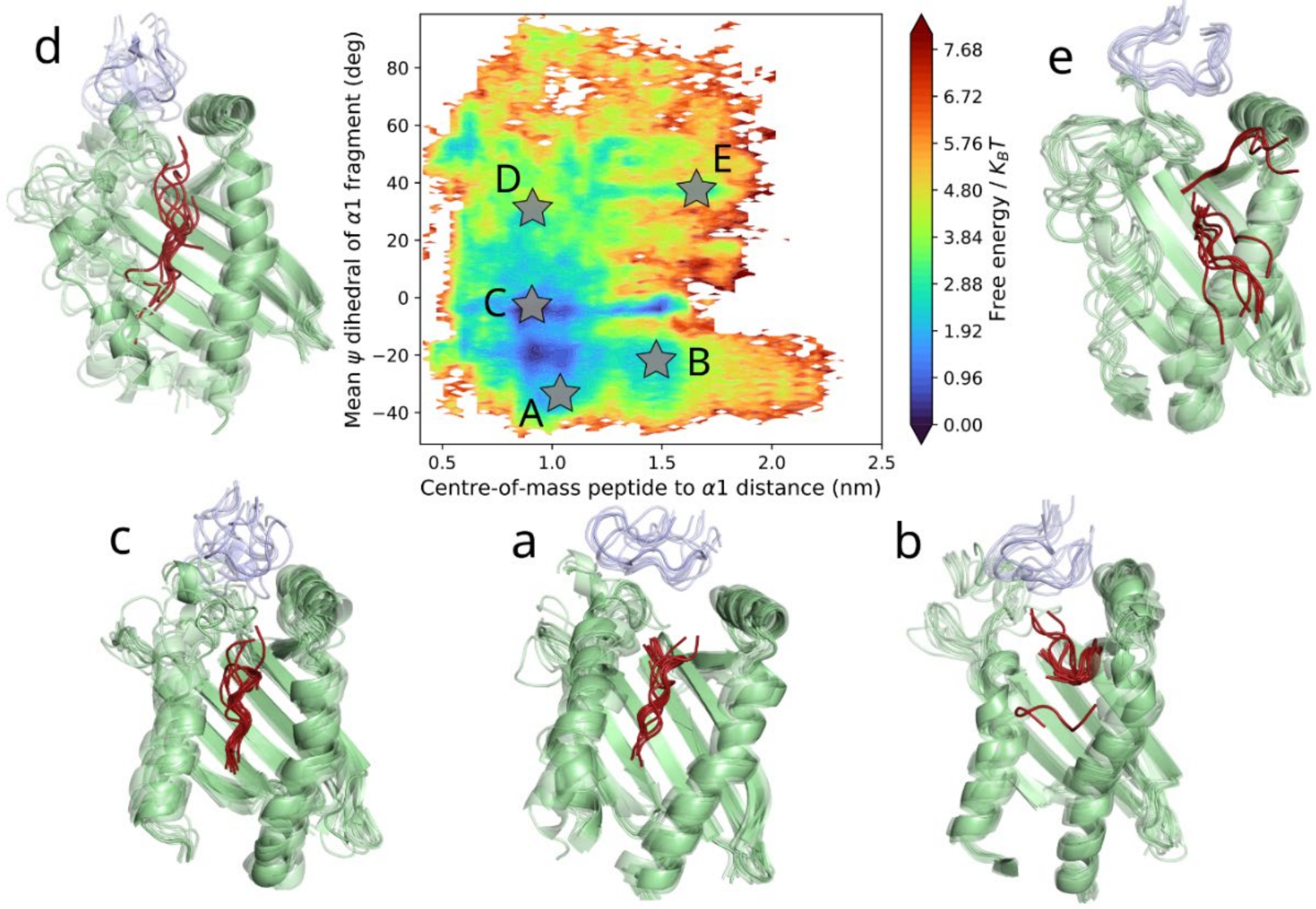

Supplementary Fig 9: MSM re-weighted Free Energy Surface (FES) projected over the peptide-α1-helix centre-of-mass distance, which measures the proximity of the peptide to the residues lining the binding groove, and the mean ψ dihedral angle of the α1-helix, which estimates the helicity. Ten structures of the MHCI(Y84C)-Tsn(K16C) complex are sampled from the highlighted each metastable states, which display the α1-helix going from a formed state (**a**) to partially formed (**b**,**c**) to fully unfolded (**d**,**e**). Starting complex was state 1 of the cryoEM structures with a restrained upper disulfide bridge.

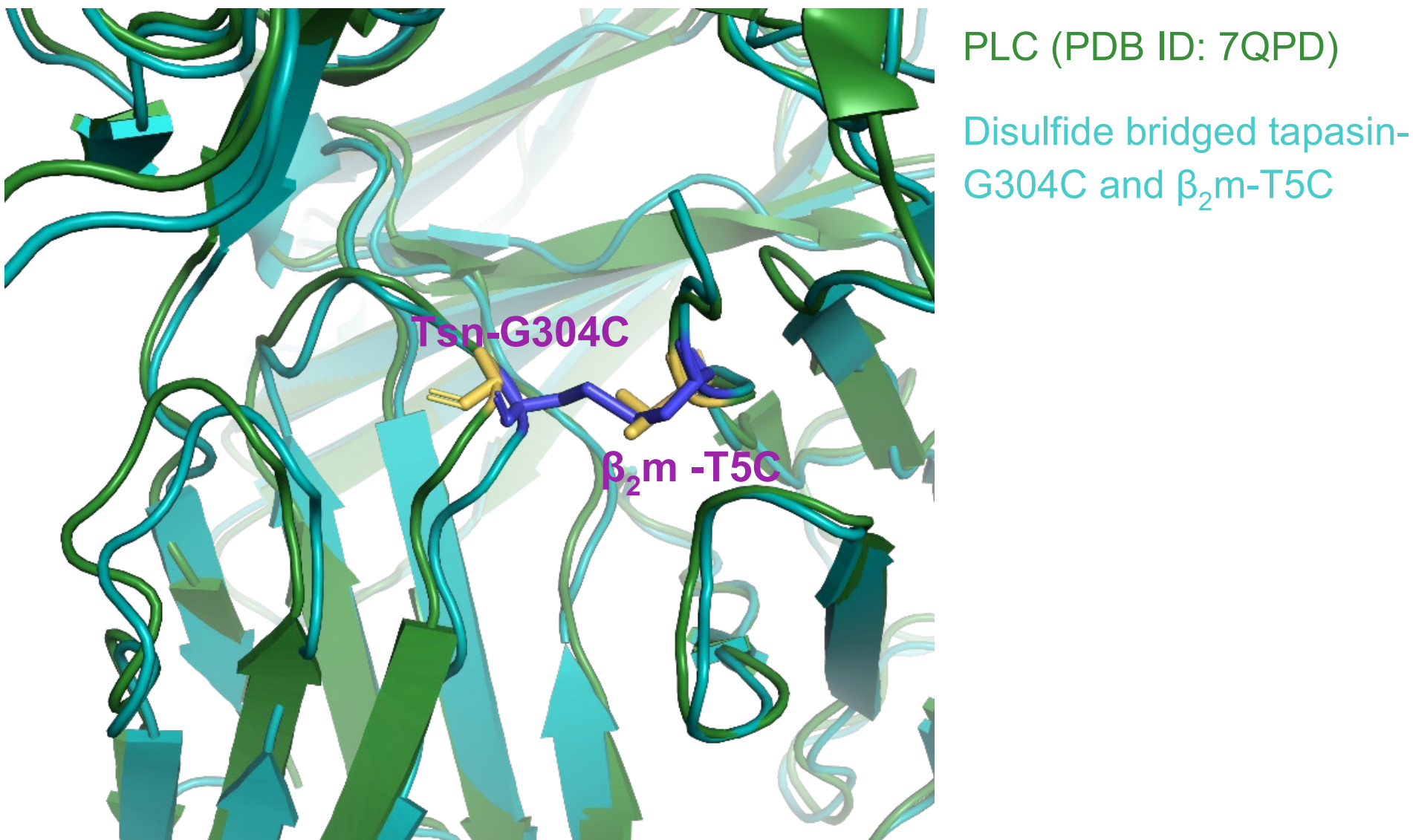

Supplementary Fig. 10: Close-up of the disulfide bridge β2m-T5C and Tsn-G304C in a superposition of our model of state 1 of the HLA-A2/Tsn variant complex with the structure of the PLC-bound MHCI (PDB ID: 7QPD[3]). Green shows the PLC structure and our model of state 1 is shown in cyan.

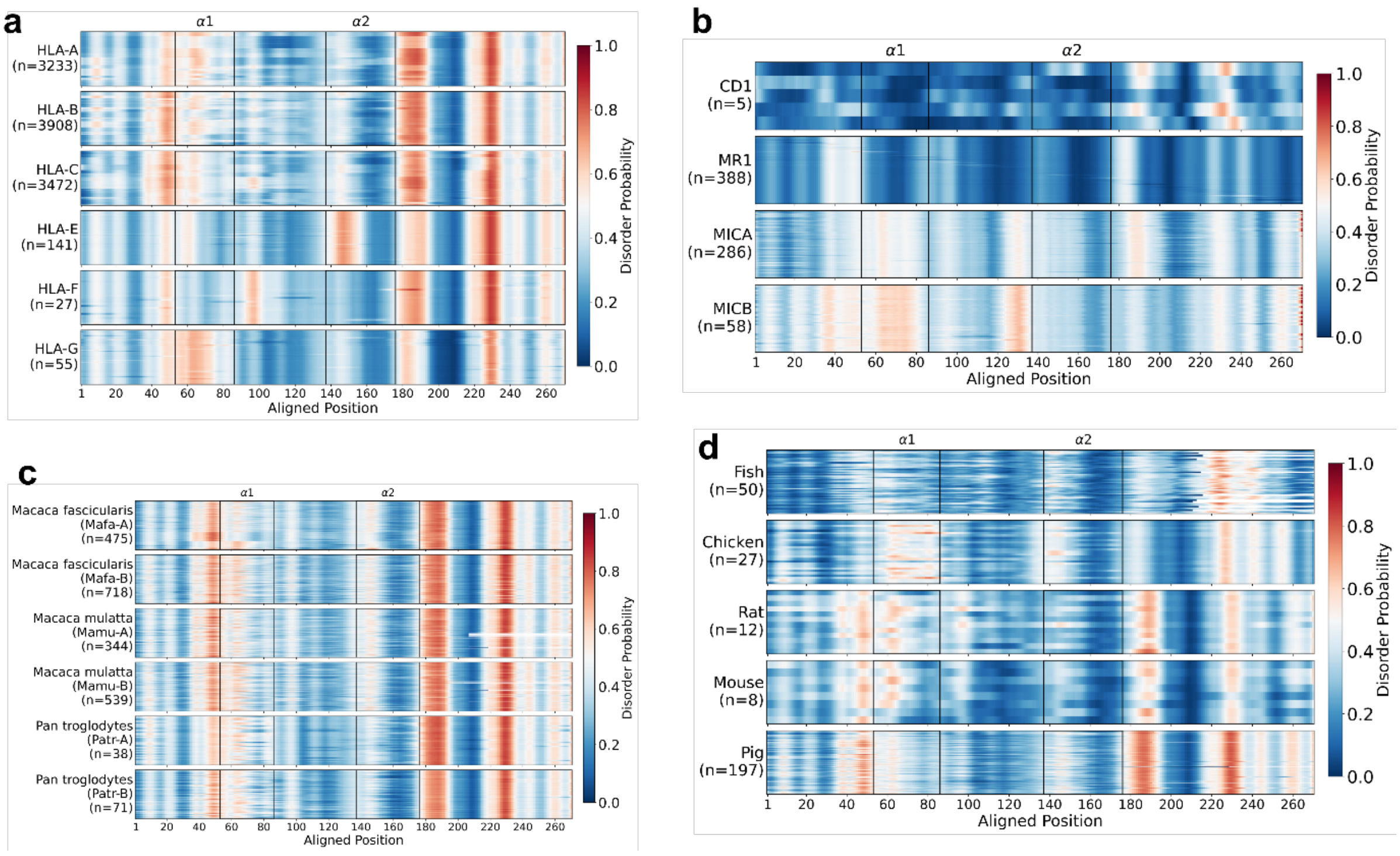

Supplementary Fig. 11: IUPred3 predictions for classical and non-classical HLA alleles as well as predictions for MHC proteins from different species. **a)** results of HLA-A, -B, -C, -E, -F and -G. **b)** results for the non-classical HLA alleles CD1, MR1, MICA and MICB. **c)** and **d)** show the predictions for different animal derived MHC proteins.

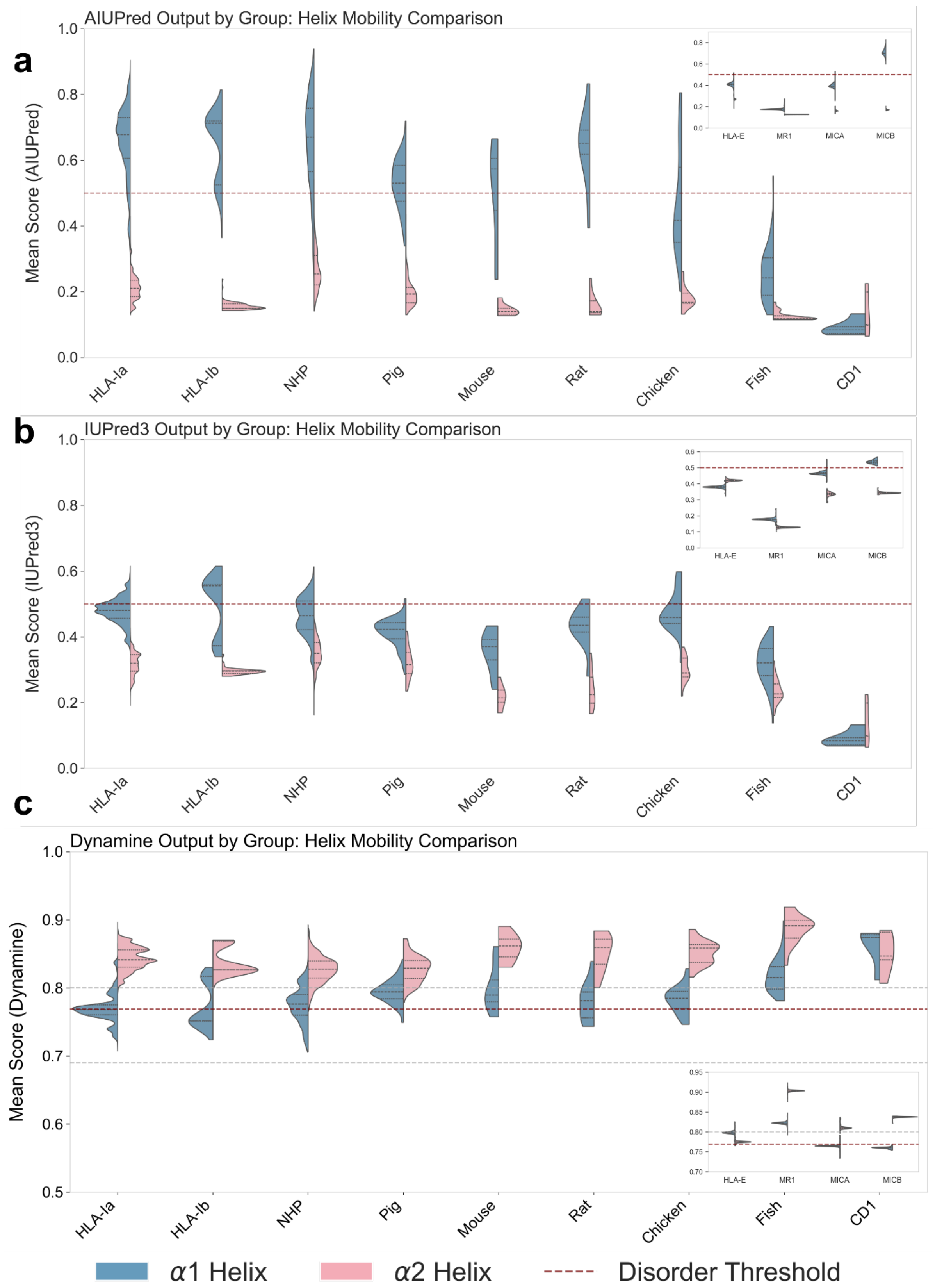
a
AIUPred Output by Group: Helix Mobility Comparison
Mean Score (AIUPred)
b
IUPred3 Output by Group: Helix Mobility Comparison
Mean Score (IUPred3)
c
Dynamine Output by Group: Helix Mobility Comparison
Mean Score (Dynamine)
HLA-Ia
HLA-Ib
NHP
Pig
Mouse
Rat
Chicken
Fish
CD1
HLA-E
MR1
MICA
MICB
α1 Helix
α2 Helix
Disorder Threshold

Supplementary Fig. 12: Violin plots for predicted disorder (AIUPred and IUPred3) and backbone rigidity (DynaMine). The results for the α1-helix (res. 53-85) are depicted in blue and for the α2-helix (137-175) in light pink. HLA-Ia contains the results for HLA-A, -B and -C, HLA-Ib for HLA-F, and -G and NHP (non-human primates, contains the results for *Macaca Mulatta*, *Pan troglodytes* and *Macaca fascicularis* (A and B alleles respectively). The dashed, red line shows in a) and b) the order vs disorder threshold of the AIUPred and IUPred predictions at 0.5. For DynaMine that threshold is at 0.769 for order vs disorder. All violins contain a line for the median as well as the 25th and 75th percentile. HLA-E, MR1, MICA and MICB are plotted in the inlay. **a)** Shows the results of the AIUPred predictions as mean scores. **b)** Shows the results of the IUPred3 prediction as mean scores and **c)** shows the results of the DynaMine predictions as mean scores. The dashed, grey lines show the thresholds for flexibility < 0.69 and rigidity > 0.8.

a

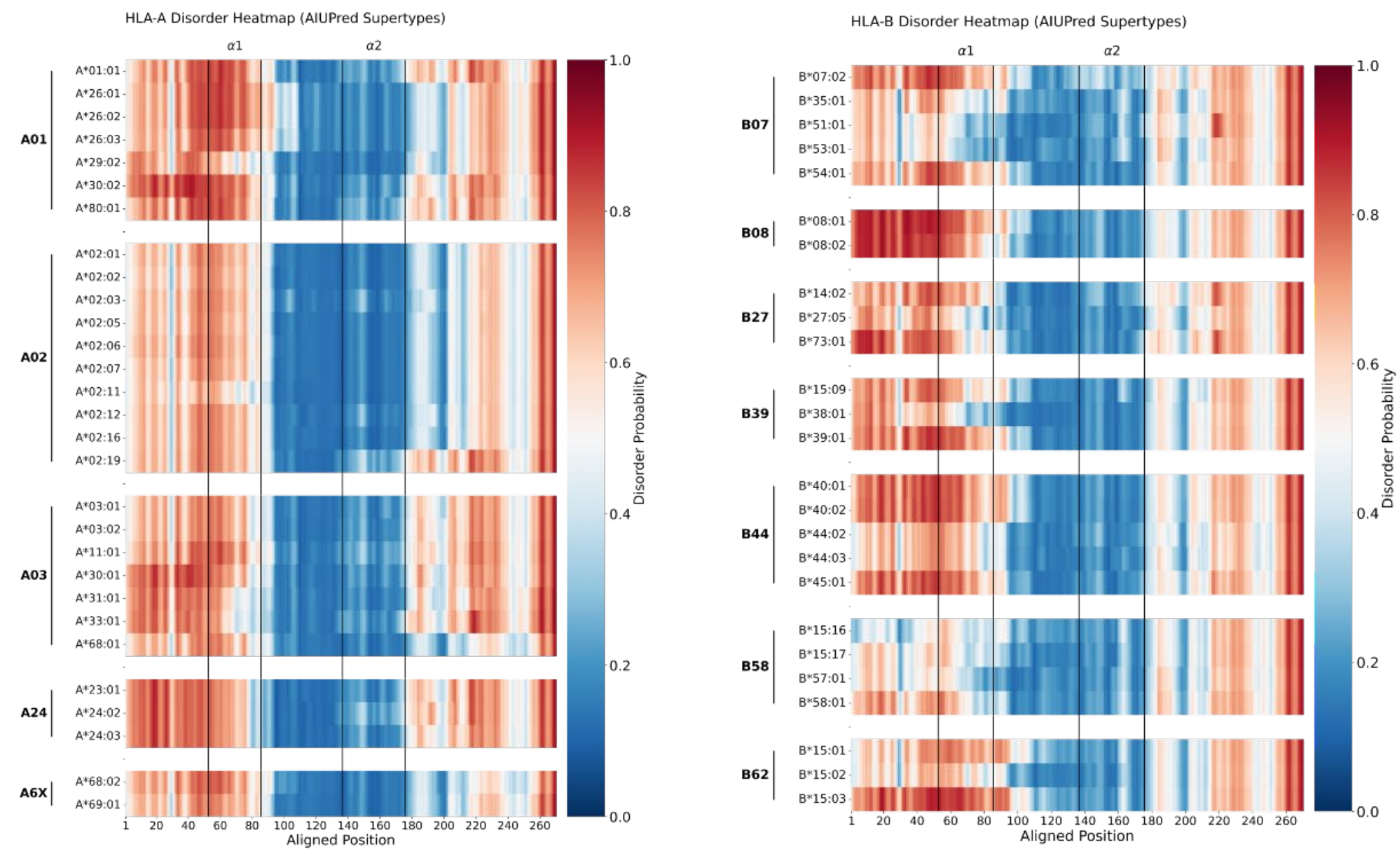

b

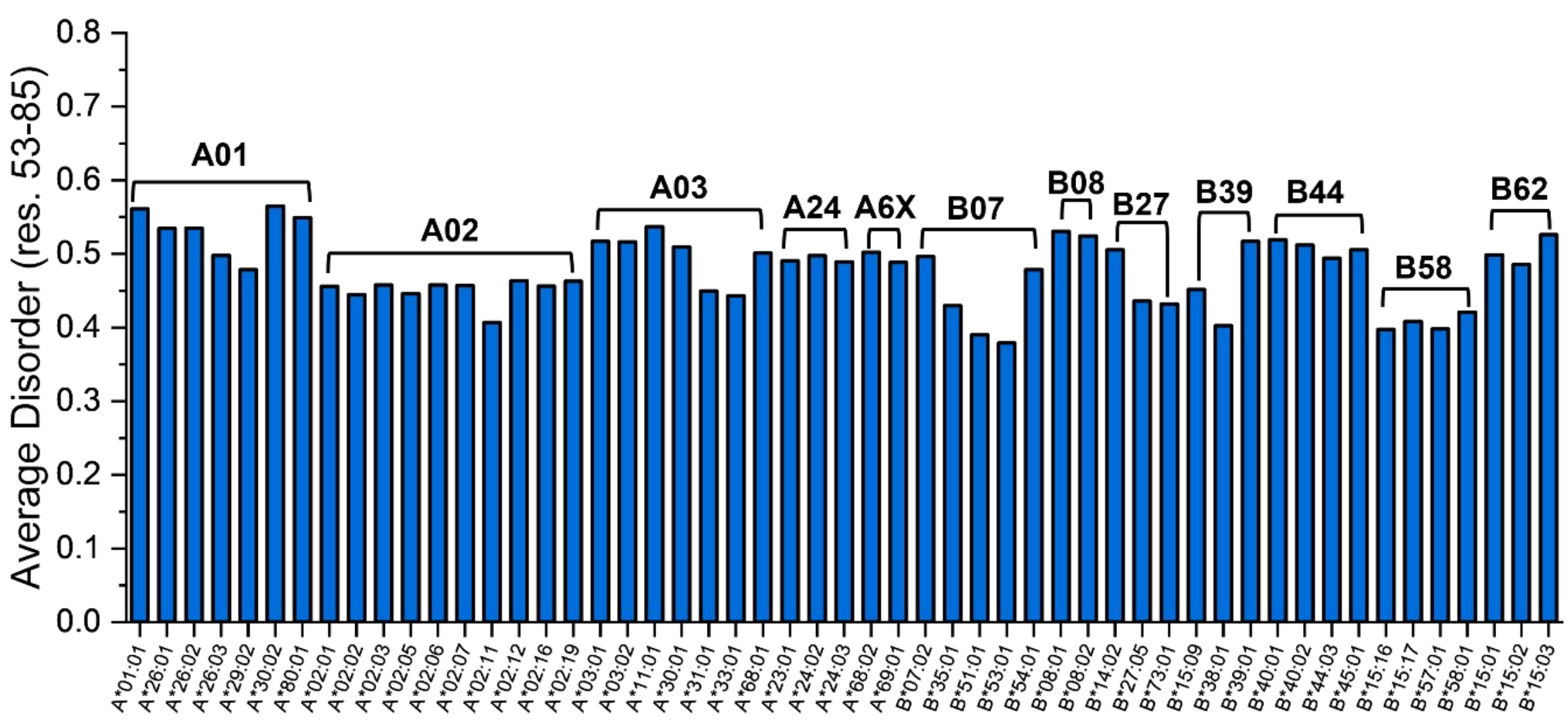

Supplementary Fig. 13: a) Heatmaps of disorder propensity sorted by supertype according to Harjanto et al.[4] **b)** Average disorder over α1-helix (res. 53-85) for alleles shown in a).

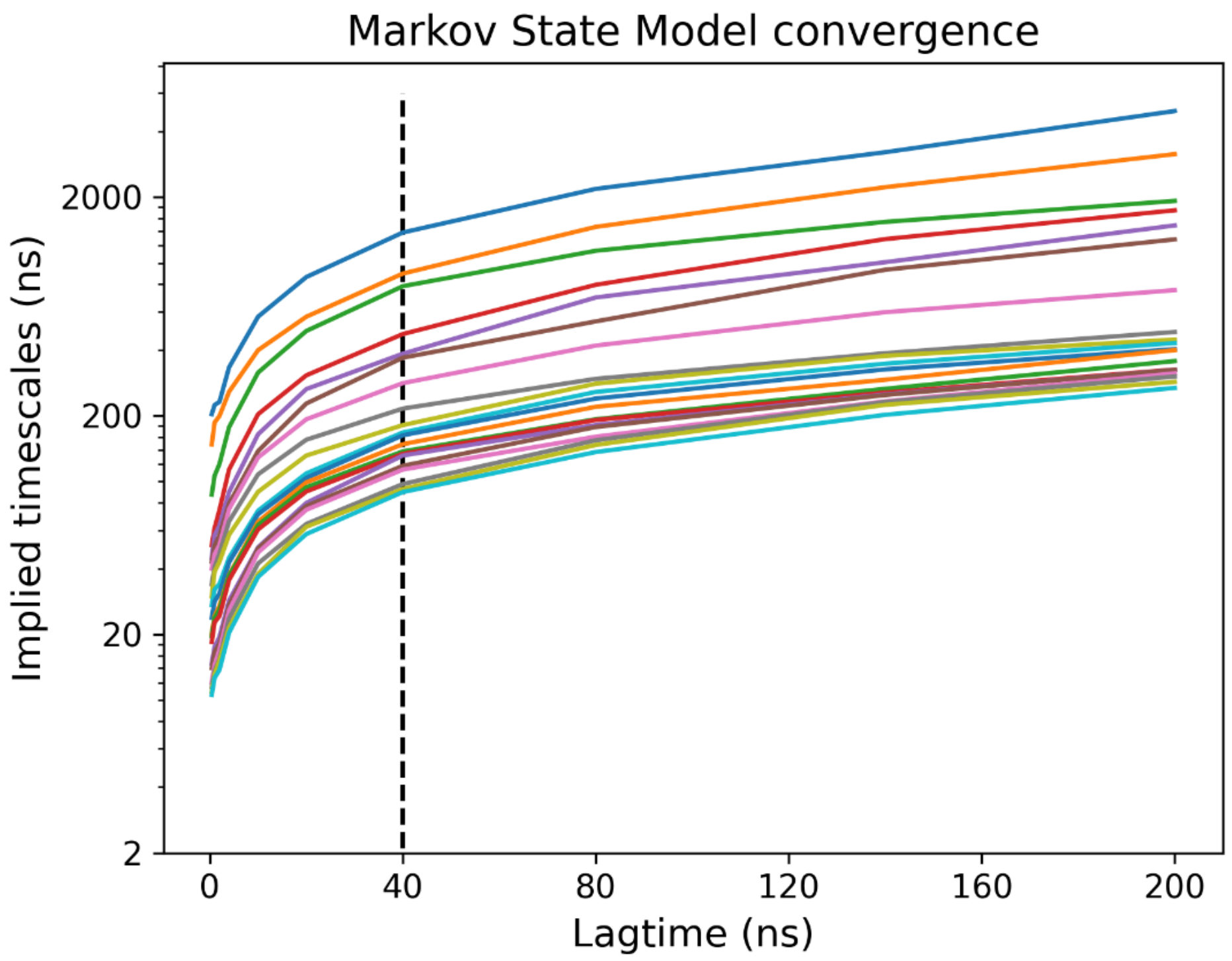

Supplementary Fig. 14: Implied timescales as function of the lagtime for the Markov state model. The dotted line is at the lagtime used to reweight the free energy surface in Suplementary Fig. 9. The partial stabilization is supportive with the convergence of the simulations.

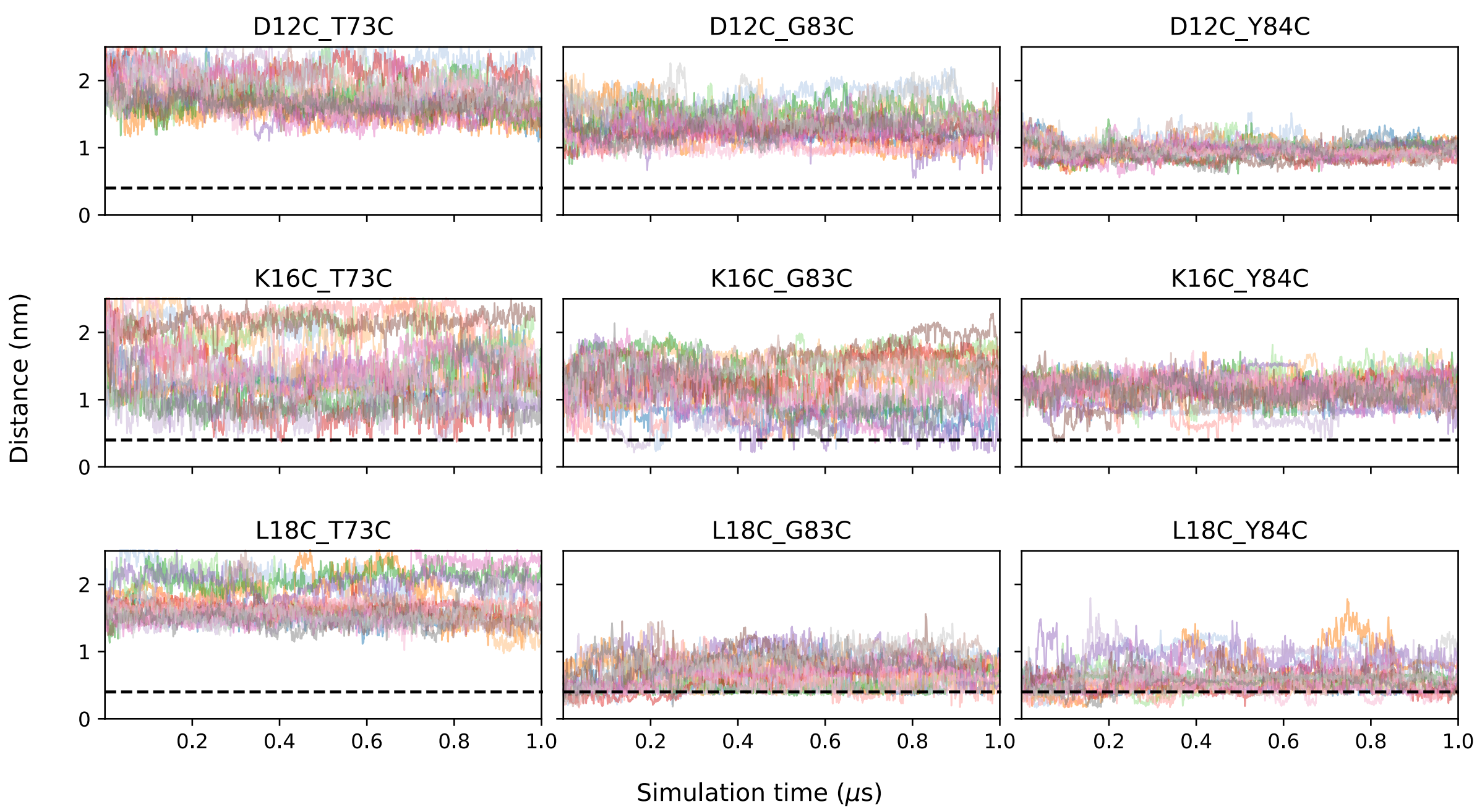

Supplementary Fig. 15: timeseries of the S-S distances shown in Fig. 2d and Sup. Fig. 3. Different color lines represent independent trajectories. The dotted line corresponds to the 4.0 Å threshold. The short and stable transitions to distances below the threshold along the trajectory are confirmatory of the possibility of generating close distances that favor a disulfide bond creation.

Supplementary Table 1: Fraction of conformations of MHCI/Tsn variant complexes where Cys-residues are closer than the 4.0 Å threshold per µs in MD simulations:

**MHC variant**	**Tapasin variant**	**Fraction of close-cysteine conformations per µs**
HLA-A*02:01-K127N/T73C	hTsn-G304C/K16C	2.39e-4
HLA-A*02:01-K127N/G83C	hTsn-G304C/K16C	8.82e-4
HLA-A*02:01-K127N/G83C	hTsn-G304C/L18C	8.48e-3
HLA-A*02:01-K127N/Y84C	hTsn-G304C/K16C	7.40e-5
HLA-A*02:01-K127N/Y84C	hTsn-G304C/L18C	1.05e-2

Supplementary Table 2: CryoEM data collection, refinement and validation statistics.

	**Complex I** (PDB 9SQN) (EMDB 55112)	**Complex II** (PDB 9SQP) (EMDB 55113)	**Complex III** (PDB 9SQO) (EMDB 55114)	**Complex IV** (PDB 9SQM) (EMDB 55111)
	State 1	State 2	State 3	State 4
**Data collection and processing**				
Microscope	FEI Titan Krios G3i			
Voltage [keV]	300			
Camera	Falcon 3EC			
Magnification	96,000	96,000	96,000	96,000
Pixel size at detector [Å/pixel]	0.819	0.819	0.819	0.819
Total electron exposure [$e^-/Å^2$]	44	42	44	42
Exposure rate [e^-/pixel/s]	0.7	0.7	0.7	0.7
Frames / exposure	33			
Defocus range [µm]	0.80 - 2			
Automation software	EPU (version 2.8.1)			
Micrographs collected	5,958	5,958	5,958	5,958
Micrographs used	5,699	5,699	5,699	5,699
Total extracted particles	2,281,616	2,281,616	2,281,616	2,281,616
Final particles	202,843	104,975	235,772	172,457
Point-group or helical symmetry parameters	C1	C1	C1	C1
Global resolution [Å]; $FSC^{a}_{0.143}$ (unmasked / masked)	3.7 / 3.5	3.9 / 3.6	3.7 / 3.5	3.8 / 3.6
Local resolution range [Å]	1.8 – 40	2.8 - 40.0	2.7 - 40	1.8 - 40.0
Map sharpening *B* factor [$Å^2$] / (*B* factor range)	-139	-129	-152	-144
Map sharpening method	Local *B* factor			
Refinement package	PHENIX (version 1.21.2_5419); real.space.refine			
**Model composition**				
Non-hydrogen atoms	9,786	9,666	9,584	9,384
Heavy chain residues	272	266	244	227
β_2m residues	99	99	99	99
Peptide residues	7	2	7	4

Tapasin residues	381	377	381	381
ERP60 residues	471	469	469	469
carbohydrate molecules	5	6	6	6
**Model refinement**				
**Model-map scores**				
CC[b] (mask)	0.79	0.78	0.80	0.81
CC (volume)	0.78	0.76	0.79	0.79
**Average *B* factors [Å^2]**				
Overall	139	155	179	167
Heavy chain	112	145	163	152
β_2m	104	117	148	142
Peptide	97	96	171	148
Tapasin	104	205	166	126
ERP60	191	288	205	212
carbohydrate molecules	135	143	168	163
**Rmsd[c] from ideal values**				
Bond lengths [Å]	0.003	0.002	0.002	0.002
Bond angles [°]	0.611	0.601	0.600	0.599
**Validation[d]**				
MolProbity score	1.84	1.97	1.95	1.97
CaBLAM outliers [%]	3.2	2.6	2.4	2.9
Clashscore	11.2	10.5	10.2	10.9
Poor rotamers [%]	1.0	1.7	1.7	1.6
Cβ deviations	0	0	0	0
EMRinger score	1.93	1.54	1.64	1.29
**Ramachandran plot**				
Favored [%]	96.0	96.3	96.3	96.2
Allowed [%]	4.0	3.7	3.6	3.8
Outliers [%]	0.0	0.0	0.1	0.0
**Ramachandran *Z*-score (rmsd)**				
Whole	0.59 (0.25)	0.61 (0.25)	0.71 (0.26)	0.78 (0.10)
Helix	2.09 (0.34)	1.71 (0.34)	1.84 (0.36)	1.15 (0.08)
Sheet	0.71 (0.27)	0.65 (0.28)	0.90 (0.28)	0.32 (0.19)
Loop	-0.52 (0.26)	-0.26 (0.27)	-0.35 (0.28)	-0.39 (0.13)

[a] FSC, Fourier shell correlation
[b] CC, correlation coefficient
[c] Rmsd, root-mean-square deviation
[d] Using MolProbity[5]

Supplementary Table 3: MHC and tapasin mutants used in this study.

MHC variants	Tapasin variants
HLA-A*02:01-K127N/Y84C	hTsn-WT
HLA-A*02:01-K127N/T73C	hTsn-G304C
HLA-A*02:01-K127N/G83C	hTsn-G304C/K16C
β_2m-T5C	hTsn-G304C/D12C
	hTsn-G304C/L18C

References

1. DeLano, W. L. Pymol: An open-source molecular graphics tool. *CCP4 Newsl. protein crystallogr* **40**, 82-92 (2002).
2. Meng, E. C. *et al.* UCSF ChimeraX: Tools for structure building and analysis. *Protein Sci* **32**, e4792 (2023). https://doi.org/10.1002/pro.4792
3. Domnick, A. *et al.* Molecular basis of MHC I quality control in the peptide loading complex. *Nat Commun* **13**, 4701 (2022). https://doi.org/10.1038/s41467-022-32384-z
4. Ebrahimi-Nik, H. *et al.* Reversion analysis reveals the in vivo immunogenicity of a poorly MHC I-binding cancer neoepitope. *Nature communications* **12**, 6423 (2021).
5. Williams, C. J. *et al.* MolProbity: More and better reference data for improved all-atom structure validation. *Protein Sci* **27**, 293-315 (2018). https://doi.org/10.1002/pro.3330
