# Supplementary material for: Dynamic states of an MHC class I molecule during peptide exchange
Source: Nat Commun. 2026 Sep 10;17:9697. doi: 10.1038/s41467-026-77731-6 (PMC13562589; doi:10.1038/s41467-026-77731-6)
Supplement: Supplementary file 2 [file 41467_2026_77731_MOESM2_ESM.pdf]

Peer Review File

Dynamic states of an MHC class I molecule during peptide exchange

Corresponding Author: Professor Christian Freund

This file contains all reviewer reports in order by version, followed by all author rebuttals in order by version.

Version 0:

Reviewer comments:

Reviewer #1

(Remarks to the Author)
Mitlehner and colleagues describe a study of the role of protein dynamics in the loading and quality control of peptides in class I MHC proteins. This is an important area of study giving the importance of peptide selection in various processes of cellular immunity. A particularly intriguing finding is the partial unfolding of the alpha 1 helix during the process with tapasin bound, which can seemingly act like a “door” for peptide exchange. A strength of the manuscript is the biochemical characterization of the various disulfide-bonded, trapped complexes necessary to stabilize the Tsn-bound complex, to include monitoring of complex formation, peptide exchange kinetics, and thermostability. Moreover, the simple yet elegant experiments with the cysteine mutations is a nice in solution validation that dynamical states are in fact important, as otherwise the various complexes could not form. I found the data and story compelling, and the surprising finding of the partially unfolded alpha 1 helix is really quite intriguing and not at all that I would have expected. I have some suggestions for what otherwise appears to be a strong manuscript and intriguing story.

1) There are repeated references (here, as well as in other papers in the field) that the major role of the PLC, including Tsn, etc. is to ensure high affinity peptides are bound. But there are known examples of weak binding peptides that are naturally processed, presented, and immunogenic – shared tumor antigens, for example with suboptimal anchors at p2. Have the authors thought how this might occur? Could the various states they hypothesize that might exist – partially unfolded helix, partially dissociated peptide, etc., somehow allow weak binders to make it through?

2) I found it surprising that MD is used to demonstrate that the disulfide bonds could form, but there is no MD in support of a partially unfolded helix. While it seems unlikely that traditional MD alone on a “regular” complex with Tsn would show helical unfolding, have the authors done any simulations beginning with partially folded states?

3) In free pMHC complexes with good binding peptides, it is the alpha2 helix that often shows dynamic character. This is often in the somewhat unstructured linker region preceding the a2-1 helix, and if anyone would have asked me what part is likely to unfold to allow QC in the PLC, I would have said the a2 helix. The authors might want to comment on this. Relatedly, the disorder predictions clearly suggest a propensity for the a1 and not the a2 helix to be disordered. The implication then is that with peptides bound and the pMHC complex released, the peptide acts as “glue” holding the a1 helix together. Some points on this might be worth mentioning.

4) With the discussion on polymorphisms, it wouldn’t be difficult at all for the authors to run the disorder predictors on various HLA polymorphic sequences. As the authors presume, it would be fascinating to see, for example, if different superfamilies display differences, and if members within superfamilies show differences, and this could very easily be added to the paper. This could also intersect with the concept of “specialists” vs. “generalists” that Jim Kaufman has discussed.

Reviewer #2

(Remarks to the Author)
Mitlehner et al report unexpected conformational flexibility in the MHC-I / tapasin interaction that allows for formation of disulfide bonds across cysteine residues engineered into the MHC-I a1-helix and tapasin editing loop despite large distances between these residues in known crystal and cryo-EM structures of MHC-I / tapasin complexes. Following up on

this observation, they performed molecular dynamics studies showing substantial conformational flexibility in the MHC-1 a1 helix that could explain the disulfide bonding observed in vitro. Cryo-EM structures of disulfide trapped complexes revealed a variety of states with missing density for bound peptide, a1-helix, or both. These structures were interpreted as intermediates in the tapasin-mediated peptide exchange reaction, leading to a novel model of peptide exchange, with a key intermediate having a tapasin-stabilized a2-helix but dynamic a1-helix that could act as a peptide-binding gatekeeper near the C-terminal side of the peptide binding site. This differs from the canonical role of tapasin as competing with peptide for MHC binding site in the F pocket, and gives rise to a new model for the mechanism of tapasin-mediated peptide exchange. In this new model tapasin serves to stabilize a transient exchange-competent or peptide-free conformation in addition to its previously defined role in competing with peptide for access to the MHC-I F pocket. This work expands on a recent paper from Nick Sgourakis's lab showing similar destabilization of the C-terminal end of the MHC-I a1-helix in the presence of TAPBPR, a chaperone related to tapasin and thought to edit peptides by a similar mechanism. A rudimentary evolutionary analysis indicated that predicted disorder in the MHC-I a-1 helix is characteristic of peptide-binding but not non-peptide-binding MHC-I homologs, and appears to have evolved with the split between mammals and birds.

Overall, the work provides an interesting new perspective on MHC-I / tapasin dynamics and contributes to evolving understanding of the molecular mechanism of peptide selection by MHC-I molecules. The experimental and computational studies are clearly presented and support the conclusions drawn although several minor inaccuracies and omissions listed below detract from the otherwise clear and compelling manuscript.

Minor points:
p 5 Fig 1b: "indicating that a time of 24 hours is required for the reaction to be complete" should be ">5hr is required for the complex to form" or something similar unless intermediate time points are available.

p. 6. For clarity, the description of molecular dynamics of the cysteine mutants should state that the proteins were modeled without disulfide bond between introduced cysteines (if that were the case).

The multiplicity of hands in the second paragraph of p10 is confusing.

p.11 paragraph 3. The entertaining state for which aY171 hydrogen bonds to the peptide N-terminus is not mentioned. Is this state 3?

The differences between AIUPred and IUPred3 and what can be learned from each analysis should be described.

Are there any differences in predicted disorder between "generalist" and "specialist" chicken MHC-I alleles as classified by Jim Kaufman (ex. Front. Immunol 2022 https://doi.org/10.3389/fimmu.2022.886672).

Is anything known about bony fish MHC-I peptide preferences that might related to dynamic differences observed here relative to mammalian MHC-I proteins?

In Fig 1C the figure or legend should indicate which of the numbered bands from panel B is shown.

In Fig 2 panels should be labeled if they correspond to particular states 1-4 as shown in sup Fig 8 and described in the text. Same for sup Fig 7.

Sup. Fig 2 could benefit from a few bands being annotated as in Fig 1b.

The video would be improved by some labeling or key to indicate which domain is which, and a description of what is being represented in the figure. Is this a single complex with density contours, or a continuum of structural states, or something else?

Reviewer #3

(Remarks to the Author)
This manuscript explores the changes in the binding groove of the MHC upon interacting with accessory proteins and variable-length peptide substrates.
The structure determinations and modeling were well-performed, as revealed in the PDB Validation Reports. The resolution was in the range of 3.5 to 3.6 Å.
The figures were clear. The conclusions were supported by the density maps and models. Importantly, the structural conclusions were based on features that were appropriate for the resolution of the maps, and were not overinterpreted. These included helices that were ordered or disordered, and the movement of bulky side chains like tyrosine, which are visible at this resolution.
One small point, on Page 11, last paragraph, a sentence reads:
"αY171 entertains a hydrogen bond to the N-terminal
amino group of the peptide only in state, while it moves away in the other structures
(Fig. 4f)." Based on the figure, I believe it should read "state 3"

Version 1:

Reviewer comments:

Reviewer #1

(Remarks to the Author)
The authors have responded well to my concerns. This is an intriguing story that adds quite a bit of intriguing molecular detail to loading of MHC-I.

Reviewer #2

(Remarks to the Author)
The revised manuscript addresses all of the concerns (minor) expressed in my review of the original manuscript.

Open Access This Peer Review File is licensed under a Creative Commons Attribution 4.0 International License, which permits use, sharing, adaptation, distribution and reproduction in any medium or format, as long as you give appropriate credit to the original author(s) and the source, provide a link to the Creative Commons license, and indicate if changes were made.
In cases where reviewers are anonymous, credit should be given to 'Anonymous Referee' and the source.
The images or other third party material in this Peer Review File are included in the article's Creative Commons license, unless indicated otherwise in a credit line to the material. If material is not included in the article's Creative Commons license and your intended use is not permitted by statutory regulation or exceeds the permitted use, you will need to obtain permission directly from the copyright holder.
To view a copy of this license, visit https://creativecommons.org/licenses/by/4.0/

Point-by-point response to reviewers

Reviewer #1 (Remarks to the Author):

Mitlehner and colleagues describe a study of the role of protein dynamics in the loading and quality control of peptides in class I MHC proteins. This is an important area of study giving the importance of peptide selection in various processes of cellular immunity. A particularly intriguing finding is the partial unfolding of the alpha 1 helix during the process with tapasin bound, which can seemingly act like a "door" for peptide exchange. A strength of the manuscript is the biochemical characterization of the various disulfide-bonded, trapped complexes necessary to stabilize the Tsn-bound complex, to include monitoring of complex formation, peptide exchange kinetics, and thermostability. Moreover, the simple yet elegant experiments with the cysteine mutations is a nice in solution validation that dynamical states are in fact important, as otherwise the various complexes could not form. I found the data and story compelling, and the surprising finding of the partially unfolded alpha 1 helix is really quite intriguing and not at all that I would have expected. I have some suggestions for what otherwise appears to be a strong manuscript and intriguing story.

Thank you for the encouragement.

1) There are repeated references (here, as well as in other papers in the field) that the major role of the PLC, including Tsn, etc. is to ensure high affinity peptides are bound. But there are known examples of weak binding peptides that are naturally processed, presented, and immunogenic – shared tumor antigens, for example with suboptimal anchors at p2. Have the authors thought how this might occur? Could the various states they hypothesize that might exist – partially unfolded helix, partially dissociated peptide, etc., somehow allow weak binders to make it through?

Yes, this is certainly an important aspect. While a general trend towards higher affinity binders in the presence of tapasin has been observed, it is still possible that peptides with intermediate binding affinity are bound if they display low off-rates. The a1-helical unfolding may allow such suboptimal peptides to encounter the MHC binding groove through the intermediate that we describe in Fig. 4 of the manuscript. This point is now described in the introduction and in the third paragraph of the Discussion: " The folding-unfolding transition of the α1-helix likely slows down initial peptide binding, as it was observed by kinetic measurements[36]. This allows peptides to sample many different conformations more easily and may lead to complexes that are characterized by low *on*-rates".

2) I found it surprising that MD is used to demonstrate that the disulfide bonds could form, but there is no MD in support of a partially unfolded helix. While it seems unlikely that traditional MD alone on a "regular" complex with Tsn would show helical unfolding,

have the authors done any simulations beginning with partially folded states?

Thank you for this very useful comment. We performed MD simulations using state 1 as starting point for the simulation. The results are shown in the new Supplementary Fig. 9 now and indicate a1-helix flexibility/unfolding to coincide with peptide dissociation.

3) In free pMHC complexes with good binding peptides, it is the alpha2 helix that often shows dynamic character. This is often in the somewhat unstructured linker region preceding the a2-1 helix, and if anyone would have asked me what part is likely to unfold to allow QC in the PLC, I would have said the a2 helix. The authors might want to comment on this. Relatedly, the disorder predictions clearly suggest a propensity for the a1 and not the a2 helix to be disordered. The implication then is that with peptides bound and the pMHC complex released, the peptide acts as "glue" holding the a1 helix together. Some points on this might be worth mentioning.

Yes, conformational dynamics has been observed for the a2 helix in the pMHCI complex, especially when not bound to tapasin or TAPBPR. It was suggested by the Sgourakis group that the dynamics in a2 are important for TAPBPR binding. We now comment on this in the discussion (p. 18) and cite the corresponding paper.

4) With the discussion on polymorphisms, it wouldn't be difficult at all for the authors to run the disorder predictors on various HLA polymorphic sequences. As the authors presume, it would be fascinating to see, for example, if different superfamilies display differences, and if members within superfamilies show differences, and this could very easily be added to the paper. This could also intersect with the concept of "specialists" vs. "generalists" that Jim Kaufman has discussed.

Thank you for these suggestions which prompted us to do some additional analyses.

For chicken MHC:

Upon analysis, we do not see a clear trend towards higher/lower disorder for one or the other classification (promiscuous/fastidious) and the disorder propensities do not follow a clear trend (see also answer to Reviewer 2).

For supertypes: *We used the classification of Harjanto et al. (Harjanto, S., Ng, L. F., & Tong, J. C. (2014). Clustering HLA class I superfamilies using structural interaction patterns. PloS one, 9(1), e86655.) to investigate the question how the supertype families differ from each other and if the alleles of one supertype are showing the same disorder propensities for the a1-helix. The Figure below shows the outcome of this analysis and is included as new Supplementary Fig. 13 in the manuscript.*

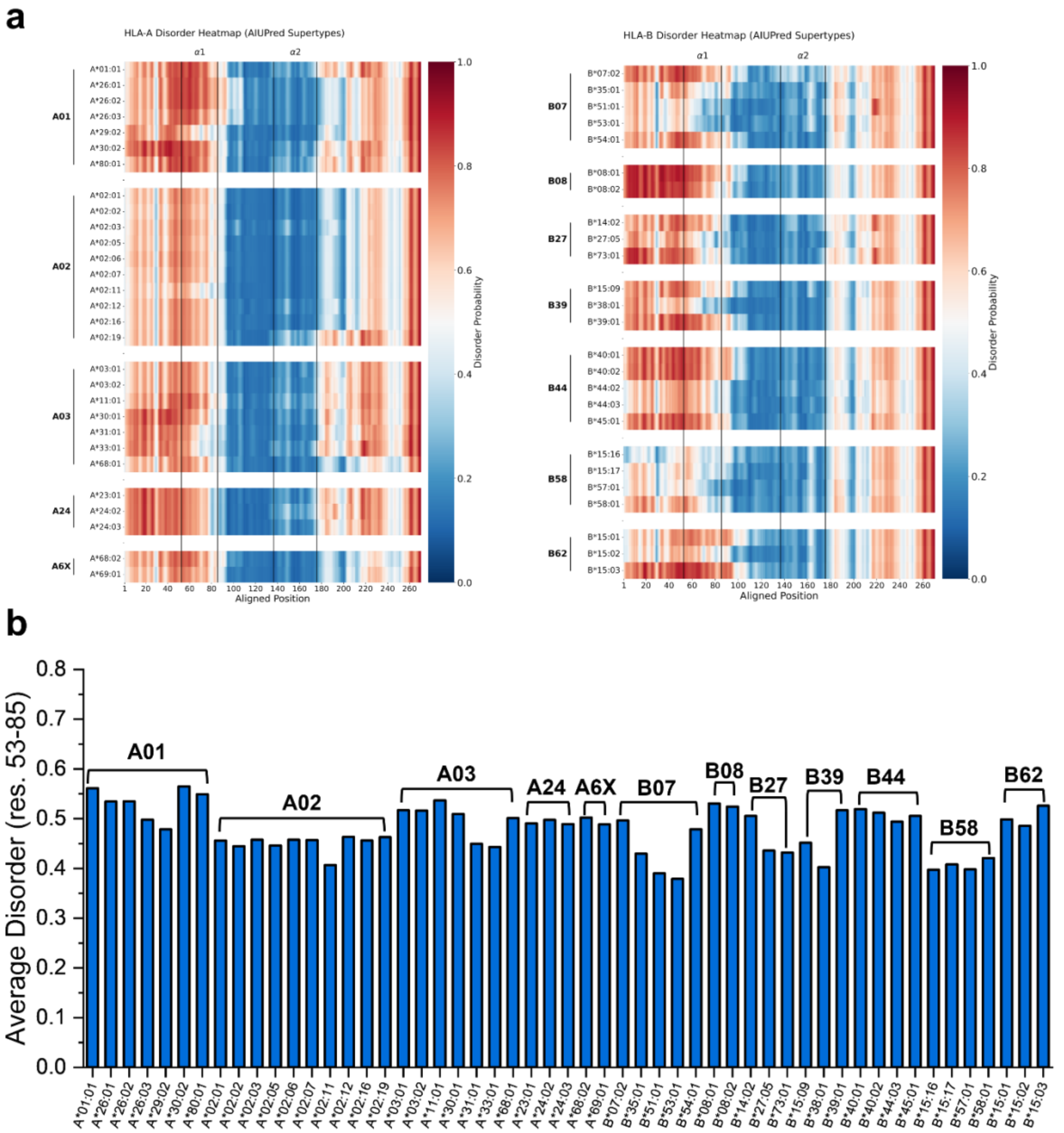

Supplementary Fig. 13: a) Heatmaps of disorder propensity sorted by supertype according to Harjanto et al. **b)** Average disorder over α1-helix (res. 53-85) for alleles shown in a).

Reviewer #2 (Remarks to the Author):

Mitlehner et al report unexpected conformational flexibility in the MHC-I / tapasin interaction that allows for formation of disulfide bonds across cysteine residues engineered into the MHC-I a1-helix and tapasin editing loop despite large distances between these residues in known crystal and cryo-EM structures of MHC-I / tapasin complexes. Following up on this observation, they performed molecular dynamics studies showing substantial conformational flexibility in the MHC-1 a1 helix that could explain the disulfide bonding observed in vitro. Cryo-EM structures of disulfide trapped

complexes revealed a variety of states with missing density for bound peptide, a1-helix, or both. These structures were interpreted as intermediates in the tapasin-mediated peptide exchange reaction, leading to a novel model of peptide exchange, with a key intermediate having a tapasin-stabilized a2-helix but dynamic a1-helix that could act as a peptide-binding gatekeeper near the C-terminal side of the peptide binding site. This differs from the canonical role of tapasin as competing with peptide for MHC binding site in the F pocket, and gives rise to a new model for the mechanism of tapasin-mediated peptide exchange. In this new model tapasin serves to stabilize a transient exchange-competent or peptide-free conformation in addition to its previously defined role in competing with peptide for access to the MHC-I F pocket. This work expands on a recent paper from Nick Sgourakis's lab showing similar destabilization of the C-terminal end of the MHC-I a1-helix in the presence of TAPBPR, a chaperone related to tapasin and thought to edit peptides by a similar mechanism. A rudimentary evolutionary analysis indicated that predicted disorder in the MHC-I a-1 helix is characteristic of peptide-binding but not non-peptide-binding MHC-I homologs, and appears to have evolved with the split between mammals and birds.

Overall, the work provides an interesting new perspective on MHC-I / tapasin dynamics and contributes to evolving understanding of the molecular mechanism of peptide selection by MHC-I molecules. The experimental and computational studies are clearly presented and support the conclusions drawn although several minor inaccuracies and omissions listed below detract from the otherwise clear and compelling manuscript.

Thank you for the overall assessment.

Minor points:

p 5 Fig 1b: "indicating that a time of 24 hours is required for the reaction to be complete" should be ">5hr is required for the complex to form" or something similar unless intermediate time points are available.

Yes, this is a correct assumption, we have changed it to >5 h.

p. 6. For clarity, the description of molecular dynamics of the cysteine mutants should state that the proteins were modeled without disulfide bond between introduced cysteines (if that were the case).

The information is now included in the legend of figure 1, Supplementary Fig. 3 and 9.

The multiplicity of hands in the second paragraph of p10 is confusing.

One set of hands was removed.

p.11 paragraph 3. The entertaining state for which aY171 hydrogen bonds to the peptide N-terminus is not mentioned. Is this state 3?

Yes, it is supposed to say state 3. Correction is included.

The differences between AIUPred and IUPred3 and what can be learned from each analysis should be described.

AIUPred presumably outperforms IUPred3 in the detection of disordered regions and employs deep learning algorithms, whilst retaining the original energy prediction. A detailed discussion of both methods is beyond the scope of this article. Despite the fact that AIUPred might have improved at predicting disordered regions, we wanted to show the results of IUPred3 which is not using any hidden features of AI but is based on a well described energy function. Also, note that although the predictions are weaker overall with IUPred3, common trends are clearly observable.

Are there any differences in predicted disorder between “generalist” and “specialist” chicken MHC-I alleles as classified by Jim Kaufman (ex. Front. Immunol 2022 https://doi.org/10.3389/fimmu.2022.886672).

This is an intriguing question, but we do not see a clear correlation between disorder propensity and the generalist/specialist classification by Kaufman and colleagues (see Figure below). We assume that at the molecular level additional factors play a role that are not revealed by the current analysis. Thus, we would rather not include the data in the manuscript, but if the reviewer thinks it is necessary, we can implement this as Suppl. Fig. as well.

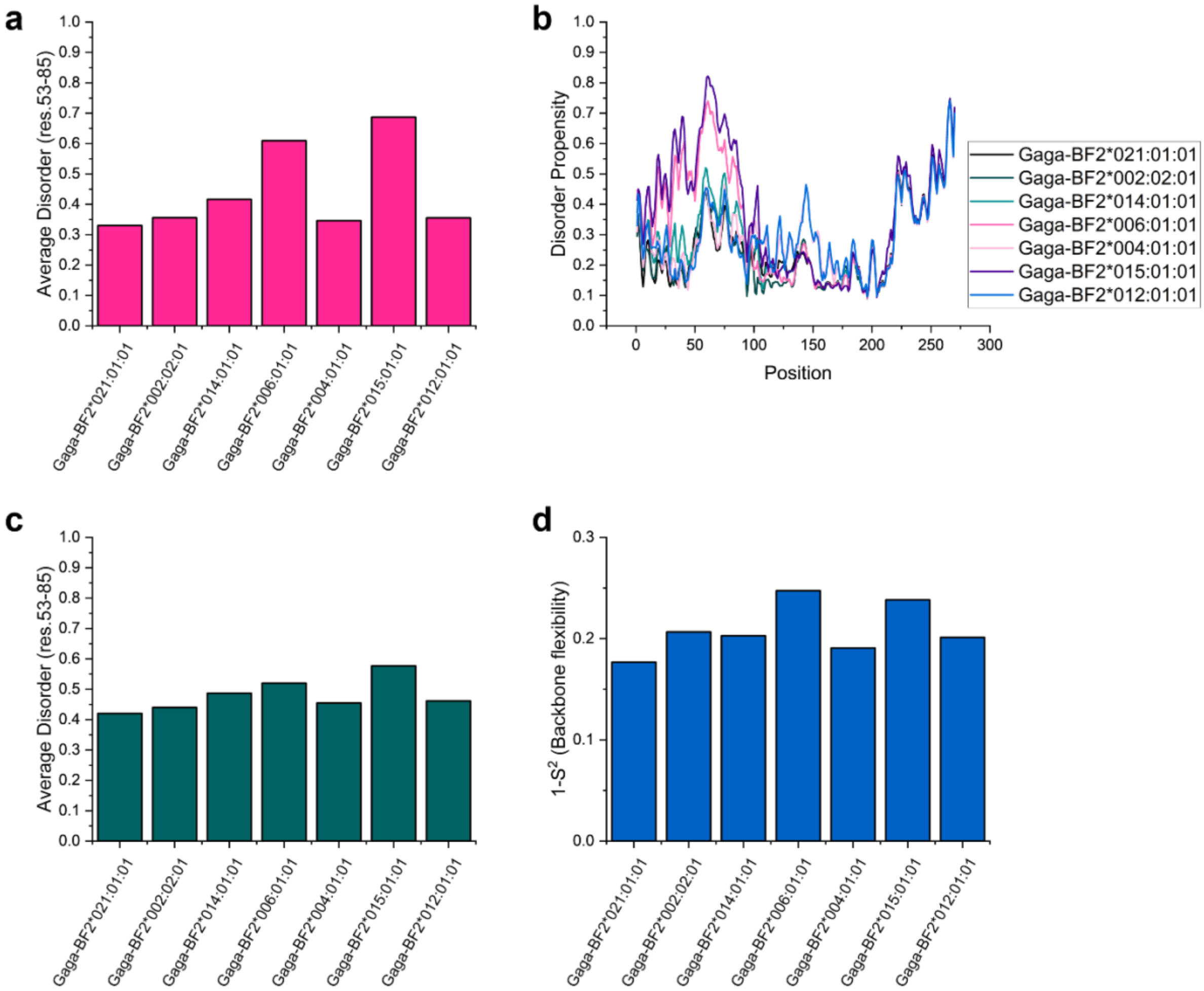

Figure chicken alleles: Average disorder propensities and 1-S^2 backbone flexibilities for chicken MHCI alleles that are named as either being promiscuous or fastidious. In all diagrams, the alleles are arranged in order from promiscuous to fastidious. All bar charts show the average prediction values for the α1-helix (res. 53–85); **a)** results from AIUPred, **c)** from IUPred3 and **d)** from Dynamine (1-S^2 backbone flexibilities). The line plot in **b)** illustrates the results for the entire sequence predicted using AIUPred.

Is anything known about bony fish MHC-I peptide preferences that might related to α2-helix dynamic differences observed here relative to mammalian MHC-I proteins?

To the best of our knowledge, no immunopeptidomic study has yet been carried out on fish and thus the data set is too sparse to allow for a statistically significant comparison.

In Fig 1C the figure or legend should indicate which of the numbered bands from panel B is shown.

Included in the figure legend.

In Fig 2 panels should be labeled if they correspond to particular states 1-4 as shown in sup Fig 8 and described in the text. Same for sup Fig 7

Now annotated the states in the panels of Fig. 3.

Sup. Fig 2 could benefit from a few bands being annotated as in Fig 1b.

Annotation was now included in the Figure.

The video would be improved by some labeling or key to indicate which domain is which, and a description of what is being represented in the figure. Is this a single complex with density contours, or a continuum of structural states, or something else?

We have now annotated the video and in the text we explicitly relate to the continuum of states that are observed.

Reviewer #3 (Remarks to the Author):

This manuscript explores the changes in the binding groove of the MHC upon interacting with accessory proteins and variable-length peptide substrates. The structure determinations and modeling were well-performed, as revealed in the PDB Validation Reports. The resolution was in the range of 3.5 to 3.6 Å. The figures were clear. The conclusions were supported by the density maps and models. Importantly, the structural conclusions were based on features that were appropriate for the resolution of the maps, and were not overinterpreted. These included helices that were ordered or disordered, and the movement of bulky side chains like tyrosine, which are visible at this resolution.

One small point, on Page 11, last paragraph, a sentence reads:

"αY171 entertains a hydrogen bond to the N-terminal amino group of the peptide only in state, while it moves away in the other structures (Fig. 4f)." Based on the figure, I believe it should read "state 3"

This was indeed to mean state 3, it is now corrected.
