# Supplementary material for: Dynamic states of an MHC class I molecule during peptide exchange
Source: Nat Commun. 2026 Sep 10;17:9697. doi: 10.1038/s41467-026-77731-6 (PMC13562589; doi:10.1038/s41467-026-77731-6)
Supplement: Supplementary file 4 [file 41467_2026_77731_MOESM4_ESM.pdf]

Description of Additional Supplementary Files

Supplementary Movie 1

Description: 3D variability analysis of cryoEM data reveals conformational dynamics within the peptide binding pocket of the HLA-A2-Tapasin-ERp57 complex.
